# Supplementary material for: Emergence to dominance: Estimating time to dominance of SARS-CoV-2 variants using nonlinear statistical models
Source: PLoS One. 2025 Apr 2;20(4):e0311459. doi: 10.1371/journal.pone.0311459 (PMC11964452; doi:10.1371/journal.pone.0311459)
Supplement: S2 File — (HTML) [file pone.0311459.s002.html]

JN1 and XBB dominance


## Table of contents

- Input data from CDC
- Dominance calculator
  - logistic
  - Weibull
  - Brain-Cousens
  - Cedergreen-Ritz-Streibig Model
  - Rootfinder - DRC
  - Generalized Additive Model
- Decay
  - weibull
  - logistic
- Compiling all Results
- For MSE calculations
- Calculating TTD
  - JN.1
    - USA
      - logistic
      - Weibull
      - GAM
      - Compile
      - MSE
    - Region 1
      - logistic
      - Weibull
      - GAM
      - Compile
      - MSE
    - Region 2
      - logistic
      - Weibull
      - GAM
      - Compile
      - MSE
    - Region 3
      - logistic
      - Weibull
      - GAM
      - Compile
      - MSE
    - Region 4
      - logistic
      - Weibull
      - GAM
      - Compile
      - MSE
    - Region 5
      - logistic
      - Weibull
      - GAM
      - Compile
      - MSE
    - Region 6
      - logistic
      - Weibull
      - GAM
      - Compile
      - MSE
    - Region 7
      - logistic
      - Weibull
      - GAM
      - Compile
      - MSE
    - Region 8
      - logistic
      - Weibull
      - GAM
      - Compile
      - MSE
    - Region 9
      - logistic
      - Weibull
      - GAM
      - Compile
      - MSE
    - Region 10
      - logistic
      - Weibull
      - GAM
      - Compile
      - MSE
  - XBB.1.5
    - USA
      - logistic
      - Weibull
      - GAM
      - Compile
      - MSE
    - Region 1
      - logistic
      - Weibull
      - GAM
      - Compile
      - MSE
    - Region 2
      - logistic
      - Weibull
      - GAM
      - Compile
      - MSE
    - Region 3
      - logistic
      - Weibull
      - GAM
      - Compile
      - MSE
    - Region 4
      - logistic
      - Weibull
      - GAM
      - Compile
      - MSE
    - Region 5
      - logistic
      - Weibull
      - GAM
      - Compile
      - MSE
    - Region 6
      - logistic
      - Weibull
      - GAM
      - Compile
      - MSE
    - Region 7
      - logistic
      - Weibull
      - GAM
      - Compile
      - MSE
    - Region 8
      - logistic
      - Weibull
      - GAM
      - Compile
      - MSE
    - Region 9
      - logistic
      - Weibull
      - GAM
      - Compile
      - MSE
    - Region 10
      - logistic
      - Weibull
      - GAM
      - Compile
      - MSE
- Finding the maximum share of the previous variant.
- Importing Weekly Testing Data
- Combining all data
- Graphs
  - AIC
  - MSE
  - TTD
    - Logistic only
  - Combined Graph for USA
    - JN.1
      - logistic
      - Weibull
      - GAM
    - XBB.1.5
      - logistic
      - Weibull
      - GAM
    - BA.5
      - logistic
      - Weibull
      - GAM
    - EG.5
      - logistic
      - Weibull
      - GAM
  - Combined Graph for Region 1
    - JN.1
      - logistic
      - Weibull
      - GAM
    - XBB.1.5
      - logistic
      - Weibull
      - GAM
  - Combined Graph for Region 2
    - JN.1
      - logistic
      - Weibull
      - GAM
    - XBB.1.5
      - logistic
      - Weibull
      - GAM
  - Combined Graph for Region 5
    - JN.1
      - logistic
      - Weibull
      - GAM
    - XBB.1.5
      - logistic
      - Weibull
      - GAM
- Combined Analysis of Logistic Estimates [ED 50]
  - USA
    - GAM
  - Region 1
    - GAM
  - Region 2
    - GAM
  - Region 3
    - GAM
  - Region 4
    - GAM
  - Region 5
    - GAM
  - Region 6
    - GAM
  - Region 7
    - GAM
  - Region 8
    - GAM
  - Region 9
    - GAM
  - Region 10
    - GAM

# JN1 and XBB dominance

Author

Miguel Fudolig

```
library(tidyverse)
```

```
── Attaching core tidyverse packages ──────────────────────── tidyverse 2.0.0 ──
✔ dplyr     1.1.4     ✔ readr     2.1.5
✔ forcats   1.0.0     ✔ stringr   1.5.1
✔ ggplot2   3.5.1     ✔ tibble    3.2.1
✔ lubridate 1.9.3     ✔ tidyr     1.3.1
✔ purrr     1.0.2     
── Conflicts ────────────────────────────────────────── tidyverse_conflicts() ──
✖ dplyr::filter() masks stats::filter()
✖ dplyr::lag()    masks stats::lag()
ℹ Use the conflicted package (<http://conflicted.r-lib.org/>) to force all conflicts to become errors
```

```
library(lubridate)
```

# Input data from CDC

```
covid <- read_csv("SARS-CoV-2_Variant_Proportions_20240507.csv") |> 
  separate(col=week_ending,into=c("week","time"),sep=" ") |> 
  separate(col=published_date,into=c("datepub","timepub"),sep=" ")
```

```
Rows: 1720451 Columns: 10
── Column specification ────────────────────────────────────────────────────────
Delimiter: ","
chr (8): usa_or_hhsregion, week_ending, variant, share_lo, nchs_or_count_fla...
dbl (2): share, share_hi

ℹ Use `spec()` to retrieve the full column specification for this data.
ℹ Specify the column types or set `show_col_types = FALSE` to quiet this message.
```

```
Warning: Expected 2 pieces. Additional pieces discarded in 1720451 rows [1, 2, 3, 4, 5,
6, 7, 8, 9, 10, 11, 12, 13, 14, 15, 16, 17, 18, 19, 20, ...].
Expected 2 pieces. Additional pieces discarded in 1720451 rows [1, 2, 3, 4, 5,
6, 7, 8, 9, 10, 11, 12, 13, 14, 15, 16, 17, 18, 19, 20, ...].
```

```
covid <- covid |> 
  mutate(week = as.Date(week,format="%m/%d/%Y"),
        datepub=as.Date(datepub,format="%m/%d/%Y")) |> 
  filter(datepub == max(datepub)) |> 
  filter(modeltype=="weighted") |> 
  mutate(region = factor(usa_or_hhsregion,levels=c("USA","1","2","3","4",
                                                        "5","6","7","8","9","10")))
                  
  

head(covid)
```

```
# A tibble: 6 × 13
  usa_or_hhsregion week       time     variant      share  share_hi share_lo    
  <chr>            <date>     <chr>    <chr>        <dbl>     <dbl> <chr>       
1 USA              2021-05-15 12:00:00 B.1.1.529 0.000142  0.000610 1.003028228…
2 USA              2021-05-15 12:00:00 B.1.617.2 0.0267    0.0329   0.021343030…
3 USA              2021-05-15 12:00:00 BA.1.1    0.000107  0.000622 2.231340204…
4 USA              2021-05-15 12:00:00 BA.2      0        NA        NULL        
5 USA              2021-05-15 12:00:00 BA.2.12.1 0        NA        NULL        
6 USA              2021-05-15 12:00:00 BA.2.75   0        NA        NULL        
# ℹ 6 more variables: nchs_or_count_flag <chr>, modeltype <chr>,
#   time_interval <chr>, datepub <date>, timepub <chr>, region <fct>
```

# Dominance calculator

## logistic

```
ndom_l <- function(r,variant_int,startdate,data=covid){
  startdate = as.Date(startdate)
  
  var_data <- data |> 
  filter(usa_or_hhsregion==r) |> 
  filter(variant %in% c(variant_int)) |> 
  mutate(t = as.numeric(week-startdate)) |> 
  arrange(t)
  

  var_data |> dplyr::select(t,share) |>slice_max(share,n=1) -> maxshare

maxtime <- maxshare[1] |> as.numeric()

lmod <- drc::drm(share~t,fct=drc::L.5(),type="continuous",data=var_data|> filter(between(t,0,maxtime)))


}
```

## Weibull

```
ndom_w <- function(r,variant_int,startdate,data=covid){
  startdate = as.Date(startdate)
  
  var_data <- data |> 
  filter(usa_or_hhsregion==r) |> 
  filter(variant %in% c(variant_int)) |> 
  mutate(t = as.numeric(week-startdate)) |> 
  arrange(t)

  var_data |> dplyr::select(t,share) |>slice_max(share,n=1) -> maxshare

maxtime <- maxshare[1] |> as.numeric()

lmod <- drc::drm(share~t,fct=drc::W1.4(),type="continuous",data=var_data|> filter(between(t,0,maxtime)))
lmod

}
```

## Brain-Cousens

```
ndom_bc <- function(r,variant_int,startdate,data=covid){
  startdate = as.Date(startdate)
  
  var_data <- data |> 
  filter(usa_or_hhsregion==r) |> 
  filter(variant %in% c(variant_int)) |> 
  mutate(t = as.numeric(week-startdate)) |> 
  arrange(t)

  var_data |> dplyr::select(t,share) |>slice_max(share,n=1) -> maxshare

maxtime <- maxshare[1] |> as.numeric()

bcmod <- drc::drm(share~t,fct=drc::BC.4(),type="continuous",data=var_data|> filter(t>=0))
# bcmod <- drc::drm(share~t,fct=drc::BC.4(),type="continuous",data=var_data|> filter(between(t,0,maxtime)))
bcmod

}

bc_function <- function(coef,t,level){
  b <- coef[1] |> as.numeric()
  d<- coef[2]|> as.numeric()
  e<- coef[3]|> as.numeric()
  f <- coef[4]|> as.numeric()
  (d+f*t)/(1+exp(b*(log(t)-log(e)))) - level
}

bc_ED <-function(Coef_ci,Level,Interval){
  uniroot(bc_function,coef=Coef_ci[,1],level=Level,interval=Interval)$root -> ci_max
  uniroot(bc_function,coef=Coef_ci[,2],level=Level,interval=Interval)$root -> ci_est
  uniroot(bc_function,coef=Coef_ci[,3],level=Level,interval=Interval)$root -> ci_min
  
  data.frame(Estimate=ci_est,
             lower=ci_min,
             upper=ci_max)
}
```

## Cedergreen-Ritz-Streibig Model

```
ndom_crs <- function(r,variant_int,startdate,data=covid){
  startdate = as.Date(startdate)
  
  var_data <- data |> 
  filter(usa_or_hhsregion==r) |> 
  filter(variant %in% c(variant_int)) |> 
  mutate(t = as.numeric(week-startdate)) |> 
  arrange(t)

  var_data |> dplyr::select(t,share) |>slice_max(share,n=1) -> maxshare

maxtime <- maxshare[1] |> as.numeric()

bcmod <- drc::drm(share~t,fct=drc::CRS.6(),type="continuous",data=var_data|> filter(t>=0))
#bcmod <- drc::drm(share~t,fct=drc::CRS.4a(),type="continuous",data=var_data|> filter(between(t,0,maxtime)))
bcmod

}

# bc_function <- function(coef,t,level){
#   b <- coef[1] |> as.numeric()
#   d<- coef[2]|> as.numeric()
#   e<- coef[3]|> as.numeric()
#   f <- coef[4]|> as.numeric()
#   (d+f*t)/(1+exp(b*(log(t)-log(e)))) - level
# }
# 
# bc_ED <-function(Coef_ci,Level,Interval){
#   uniroot(bc_function,coef=Coef_ci[,1],level=Level,interval=Interval)$root -> ci_max
#   uniroot(bc_function,coef=Coef_ci[,2],level=Level,interval=Interval)$root -> ci_est
#   uniroot(bc_function,coef=Coef_ci[,3],level=Level,interval=Interval)$root -> ci_min
#   
#   data.frame(Estimate=ci_est,
#              lower=ci_min,
#              upper=ci_max)
# }
```

## Rootfinder - DRC

```
ttd_drc <- function(x,mod1,level=0.5){
  a <-predict(mod1,newdata=data.frame(t=x))
  as.numeric(a) - level
}

drc_rootfinder <- function(mod,Range,Level=0.5){
  uniroot(ttd_drc,interval=Range,mod1=mod,level=Level)
}
```

## Generalized Additive Model

```
ndom_gam <- function(r,variant_int,startdate,data=covid){
  startdate = as.Date(startdate)
  
  var_data <- data |> 
  filter(usa_or_hhsregion==r) |> 
  filter(variant %in% c(variant_int)) |> 
  mutate(t = as.numeric(week-startdate)) |> 
  arrange(t)

  var_data |> dplyr::select(t,share) |>slice_max(share,n=1) -> maxshare

maxtime <- maxshare[1] |> as.numeric()

# lmod <- mgcv::gam(share~s(t),data=var_data |> filter(t>=0))
lmod <- mgcv::gam(share~s(t),data=var_data |> filter(between(t,0,maxtime)))
lmod

}

ttd_gam <- function(x,mod1,level=0.5){
  a <-predict(mod1,newdata=data.frame(t=x),se.fit=T,type="response")
  as.numeric(a$fit) - level
}

rootfinder <-function(mod,Range,Level=0.5){
  uniroot(ttd_gam,interval=Range,mod1=mod,level=Level)
}
```

# Decay

## weibull

```
decay_w <- function(r,variant_int,startdate,data=covid){
  startdate = as.Date(startdate)
  
  var_data <- data |> 
  filter(usa_or_hhsregion==r) |> 
  filter(variant %in% c(variant_int)) |> 
  mutate(t = as.numeric(week-startdate)) |> 
  arrange(t)

  var_data |> dplyr::select(t,share) |>slice_max(share,n=1) -> maxshare

maxtime <- maxshare[1] |> as.numeric()

var_data <- var_data |> mutate(tdecay = t-maxtime)

wmod <- drc::drm(share~t,fct=drc::W1.4(),type="continuous",data=var_data|> filter(tdecay >= 0))
wmod


}
```

## logistic

```
decay_l <- function(r,variant_int,startdate,data=covid){
  startdate = as.Date(startdate)
  
  var_data <- data |> 
  filter(usa_or_hhsregion==r) |> 
  filter(variant %in% c(variant_int)) |> 
  mutate(t = as.numeric(week-startdate)) |> 
  arrange(t)

  var_data |> dplyr::select(t,share) |>slice_max(share,n=1) -> maxshare

maxtime <- maxshare[1] |> as.numeric()

var_data <- var_data |> mutate(tdecay = t-maxtime)

lmod <- drc::drm(share~t,fct=drc::logistic(),type="continuous",data=var_data|> filter(tdecay >= 0))
lmod

# llmod <- drc::drm(share~t,fct=drc::LL.5(),type="continuous",data=eg5 |> filter(between(t,0,maxtime)))
# #plot(eg5_llmod) 
# 
# wmod <- drc::drm(share~t,fct=drc::W1.4(),type="continuous",data=eg5 |> filter(between(t,0,maxtime)))
# #plot(eg5_wmod)

}
```

# Compiling all Results

```
ttd <- function(modl, modw,modg,Region,variant_int){
  maxs<- covid |> 
  filter(usa_or_hhsregion==Region) |> 
  filter(variant %in% c(variant_int)) |> 
  mutate(t = as.numeric(week-startdate)) |> 
  arrange(t) |> 
    slice_max(share,n=1) |> 
    dplyr::select(t,share) 
  
  ShareMax <-as.numeric(maxs[,2]) 
  maxshare <- if_else(ShareMax < 0.5,1,ShareMax)
  maxtime <- as.numeric(maxs[,1])
  data.frame(Region=Region,
             Variant=variant_int,
             AIC_l = AIC(modl),
             AIC_w = AIC(modw),
             AIC_g = AIC(modg),
             g50 = rootfinder(mod = modg,Range=c(1,maxtime))$root,
             l50= drc_rootfinder(mod=modl,Range=c(1,maxtime))$root,
             w50=drc_rootfinder(mod=modw,Range=c(1,maxtime))$root,
             l25= drc_rootfinder(mod=modl,Range=c(1,maxtime),Level=0.25)$root,
             w25= drc_rootfinder(mod=modl,Range=c(1,maxtime),Level=0.25)$root,
             g25= rootfinder(mod=modg,Range=c(1,maxtime),Level=0.25)$root,
             maxshare=ShareMax
)
}
```

# For MSE calculations

```
MSE_JN1 <- data.frame(Variant=NA,
                      Region=NA,
                      t=NA,
                    share=NA,
                    y=NA,
                    logistic=NA,
                    weibull=NA)
MSE_XBB <- data.frame(Variant=NA,
                      Region=NA,
                      t=NA,
                    share=NA,
                    y=NA,
                    logistic=NA,
                    weibull=NA)
```

# Calculating TTD

## JN.1

```
startdate <- as.Date("2023-09-02")
```

### USA

#### logistic

```
lmod <- ndom_l(r="USA",
               variant_int="JN.1",
               startdate=startdate)

summary(lmod)
```

```
Model fitted: Generalised logistic (ED50 as parameter) (5 parms)

Parameter estimates:

                 Estimate  Std. Error  t-value   p-value    
b:(Intercept) -8.1006e-02  4.0044e-03 -20.2293 1.807e-07 ***
c:(Intercept) -8.4659e-04  1.8258e-03  -0.4637     0.657    
d:(Intercept)  8.4544e-01  6.5711e-03 128.6606 4.520e-13 ***
e:(Intercept)  1.1396e+02  1.7564e+00  64.8835 5.427e-11 ***
f:(Intercept)  9.3433e-01  1.0064e-01   9.2840 3.484e-05 ***
---
Signif. codes:  0 '***' 0.001 '**' 0.01 '*' 0.05 '.' 0.1 ' ' 1

Residual standard error:

 0.003515516 (7 degrees of freedom)
```

```
plot(lmod)
```

```
AIC(lmod)
```

```
[1] -96.02709
```

#### Weibull

```
wmod <- ndom_w(r="USA",
               variant_int="JN.1",
               startdate=startdate)

summary(wmod)
```

```
Model fitted: Weibull (type 1) (4 parms)

Parameter estimates:

                 Estimate  Std. Error t-value   p-value    
b:(Intercept) -4.1559e+00  6.1357e-01 -6.7733 0.0001416 ***
c:(Intercept)  8.7682e-04  8.5094e-03  0.1030 0.9204662    
d:(Intercept)  1.0649e+00  9.2951e-02 11.4562 3.050e-06 ***
e:(Intercept)  1.0967e+02  2.7147e+00 40.3976 1.551e-10 ***
---
Signif. codes:  0 '***' 0.001 '**' 0.01 '*' 0.05 '.' 0.1 ' ' 1

Residual standard error:

 0.02016745 (8 degrees of freedom)
```

```
plot(wmod)
```

```
AIC(wmod)
```

```
[1] -54.49951
```

#### GAM

```
Region<- "USA"
```

```
gmod <- ndom_gam(r=Region,
               variant_int="JN.1",
               startdate=startdate)

summary(gmod)
```

```
Family: gaussian 
Link function: identity 

Formula:
share ~ s(t)

Parametric coefficients:
             Estimate Std. Error t value Pr(>|t|)    
(Intercept) 0.2442248  0.0009346   261.3 1.91e-06 ***
---
Signif. codes:  0 '***' 0.001 '**' 0.01 '*' 0.05 '.' 0.1 ' ' 1

Approximate significance of smooth terms:
      edf Ref.df     F  p-value    
s(t) 8.58   8.95 12088 8.03e-05 ***
---
Signif. codes:  0 '***' 0.001 '**' 0.01 '*' 0.05 '.' 0.1 ' ' 1

R-sq.(adj) =      1   Deviance explained =  100%
GCV = 5.1977e-05  Scale est. = 1.0483e-05  n = 12
```

```
plot(gmod)
```

```
AIC(gmod)
```

```
[1] -101.5883
```

```
rootfinder(mod = gmod,Range=c(1,150))
```

```
$root
[1] 117.6403

$f.root
[1] -2.193257e-09

$iter
[1] 7

$init.it
[1] NA

$estim.prec
[1] 6.103516e-05
```

#### Compile

```
ttd(modl=lmod,
    modw=wmod,
    modg=gmod,
    Region=Region,
    variant = "JN.1")->ttdju
```

#### MSE

```
Variant<- "JN.1"
methodsu<-data.frame(Variant=Variant,
                     Region=Region,
                     t=gmod$model$t,
                    share=gmod$model$share,
                    y=gmod$fitted.values,
                    logistic=c(lmod$predres[,1]),
                    weibull=c(wmod$predres[,1])
                    ) 

MSE_JN1<- bind_rows(MSE_JN1,methodsu)
```

### Region 1

#### logistic

```
lmod <- ndom_l(r="1",
               variant_int="JN.1",
               startdate=startdate)

summary(lmod)
```

```
Model fitted: Generalised logistic (ED50 as parameter) (5 parms)

Parameter estimates:

                 Estimate  Std. Error t-value   p-value    
b:(Intercept) -1.4274e-01  2.2281e-02 -6.4064  0.000365 ***
c:(Intercept) -9.6013e-04  6.5147e-03 -0.1474  0.886988    
d:(Intercept)  8.5009e-01  1.1831e-02 71.8503 2.660e-11 ***
e:(Intercept)  1.2093e+02  2.1013e+00 57.5505 1.255e-10 ***
f:(Intercept)  4.1235e-01  9.7854e-02  4.2140  0.003966 ** 
---
Signif. codes:  0 '***' 0.001 '**' 0.01 '*' 0.05 '.' 0.1 ' ' 1

Residual standard error:

 0.01228395 (7 degrees of freedom)
```

```
plot(lmod)
```

```
AIC(lmod)
```

```
[1] -66.00051
```

#### Weibull

```
wmod <- ndom_w(r="1",
               variant_int="JN.1",
               startdate=startdate)

summary(wmod)
```

```
Model fitted: Weibull (type 1) (4 parms)

Parameter estimates:

                 Estimate  Std. Error t-value   p-value    
b:(Intercept)  -4.9823773   1.4469201 -3.4434  0.008779 ** 
c:(Intercept)   0.0053999   0.0170982  0.3158  0.760222    
d:(Intercept)   1.0312404   0.1292851  7.9765 4.460e-05 ***
e:(Intercept) 106.0617525   3.0232929 35.0815 4.769e-10 ***
---
Signif. codes:  0 '***' 0.001 '**' 0.01 '*' 0.05 '.' 0.1 ' ' 1

Residual standard error:

 0.03908719 (8 degrees of freedom)
```

```
plot(wmod)
```

```
AIC(wmod)
```

```
[1] -38.61811
```

#### GAM

```
Region<- "1"
```

```
gmod <- ndom_gam(r=Region,
               variant_int="JN.1",
               startdate=startdate)

summary(gmod)
```

```
Family: gaussian 
Link function: identity 

Formula:
share ~ s(t)

Parametric coefficients:
            Estimate Std. Error t value Pr(>|t|)    
(Intercept)  0.26824    0.00259   103.6 1.35e-05 ***
---
Signif. codes:  0 '***' 0.001 '**' 0.01 '*' 0.05 '.' 0.1 ' ' 1

Approximate significance of smooth terms:
       edf Ref.df    F p-value    
s(t) 8.505  8.932 1843 0.00054 ***
---
Signif. codes:  0 '***' 0.001 '**' 0.01 '*' 0.05 '.' 0.1 ' ' 1

R-sq.(adj) =  0.999   Deviance explained =  100%
GCV = 0.00038725  Scale est. = 8.0506e-05  n = 12
```

```
plot(gmod)
```

```
AIC(gmod)
```

```
[1] -76.90992
```

```
rootfinder(mod = gmod,Range=c(1,150))
```

```
$root
[1] 114.2051

$f.root
[1] 9.731327e-12

$iter
[1] 7

$init.it
[1] NA

$estim.prec
[1] 6.103516e-05
```

#### Compile

```
ttd(modl=lmod,
    modw=wmod,
    modg=gmod,
    Region=Region,
    variant = "JN.1")->ttdj1
```

#### MSE

```
Variant<- "JN.1"
methodsu<-data.frame(Variant=Variant,
                     Region=Region,
                     t=gmod$model$t,
                    share=gmod$model$share,
                    y=gmod$fitted.values,
                    logistic=c(lmod$predres[,1]),
                    weibull=c(wmod$predres[,1])
                    ) 

MSE_JN1<- bind_rows(MSE_JN1,methodsu)
```

### Region 2

#### logistic

```
lmod <- ndom_l(r="2",
               variant_int="JN.1",
               startdate=startdate)

summary(lmod)
```

```
Model fitted: Generalised logistic (ED50 as parameter) (5 parms)

Parameter estimates:

                 Estimate  Std. Error  t-value   p-value    
b:(Intercept) -1.2286e-01  4.6657e-03 -26.3320 2.916e-08 ***
c:(Intercept) -9.0162e-04  1.6252e-03  -0.5548    0.5963    
d:(Intercept)  8.2046e-01  2.6639e-03 307.9914 9.463e-16 ***
e:(Intercept)  1.1122e+02  7.2895e-01 152.5747 1.372e-13 ***
f:(Intercept)  5.5210e-01  3.7053e-02  14.9003 1.470e-06 ***
---
Signif. codes:  0 '***' 0.001 '**' 0.01 '*' 0.05 '.' 0.1 ' ' 1

Residual standard error:

 0.002977742 (7 degrees of freedom)
```

```
plot(lmod)
```

```
AIC(lmod)
```

```
[1] -100.0116
```

#### Weibull

```
wmod <- ndom_w(r="2",
               variant_int="JN.1",
               startdate=startdate)

summary(wmod)
```

```
Model fitted: Weibull (type 1) (4 parms)

Parameter estimates:

                Estimate Std. Error t-value   p-value    
b:(Intercept) -5.4445377  0.9499504 -5.7314 0.0004383 ***
c:(Intercept)  0.0018884  0.0131783  0.1433 0.8896011    
d:(Intercept)  0.9223787  0.0567556 16.2518 2.067e-07 ***
e:(Intercept) 97.8938017  1.7114174 57.2004 9.687e-12 ***
---
Signif. codes:  0 '***' 0.001 '**' 0.01 '*' 0.05 '.' 0.1 ' ' 1

Residual standard error:

 0.03102589 (8 degrees of freedom)
```

```
plot(wmod)
```

```
AIC(wmod)
```

```
[1] -44.16146
```

#### GAM

```
Region<- "2"
```

```
gmod <- ndom_gam(r=Region,
               variant_int="JN.1",
               startdate=startdate)

summary(gmod)
```

```
Family: gaussian 
Link function: identity 

Formula:
share ~ s(t)

Parametric coefficients:
            Estimate Std. Error t value Pr(>|t|)    
(Intercept) 0.287984   0.001731   166.4 3.96e-06 ***
---
Signif. codes:  0 '***' 0.001 '**' 0.01 '*' 0.05 '.' 0.1 ' ' 1

Approximate significance of smooth terms:
       edf Ref.df    F  p-value    
s(t) 8.496  8.929 4160 1.31e-05 ***
---
Signif. codes:  0 '***' 0.001 '**' 0.01 '*' 0.05 '.' 0.1 ' ' 1

R-sq.(adj) =      1   Deviance explained =  100%
GCV = 0.00017225  Scale est. = 3.5943e-05  n = 12
```

```
plot(gmod)
```

```
AIC(gmod)
```

```
[1] -86.56056
```

```
rootfinder(mod = gmod,Range=c(1,150))
```

```
$root
[1] 108.1952

$f.root
[1] 1.9066e-09

$iter
[1] 7

$init.it
[1] NA

$estim.prec
[1] 6.103516e-05
```

#### Compile

```
ttd(modl=lmod,
    modw=wmod,
    modg=gmod,
    Region=Region,
    variant = "JN.1")->ttdj2
```

#### MSE

```
Variant<- "JN.1"
methodsu<-data.frame(Variant=Variant,
                     Region=Region,
                     t=gmod$model$t,
                    share=gmod$model$share,
                    y=gmod$fitted.values,
                    logistic=c(lmod$predres[,1]),
                    weibull=c(wmod$predres[,1])
                    ) 

MSE_JN1<- bind_rows(MSE_JN1,methodsu)
```

### Region 3

#### logistic

```
lmod <- ndom_l(r="3",
               variant_int="JN.1",
               startdate=startdate)

summary(lmod)
```

```
Model fitted: Generalised logistic (ED50 as parameter) (5 parms)

Parameter estimates:

                 Estimate  Std. Error  t-value   p-value    
b:(Intercept) -8.5700e-02  5.0958e-03 -16.8177 6.432e-07 ***
c:(Intercept) -8.0225e-04  2.0623e-03  -0.3890 0.7088354    
d:(Intercept)  8.3084e-01  7.2946e-03 113.8977 1.060e-12 ***
e:(Intercept)  1.1444e+02  2.0244e+00  56.5293 1.422e-10 ***
f:(Intercept)  9.3081e-01  1.2112e-01   7.6853 0.0001177 ***
---
Signif. codes:  0 '***' 0.001 '**' 0.01 '*' 0.05 '.' 0.1 ' ' 1

Residual standard error:

 0.00411622 (7 degrees of freedom)
```

```
plot(lmod)
```

```
AIC(lmod)
```

```
[1] -92.24112
```

#### Weibull

```
wmod <- ndom_w(r="3",
               variant_int="JN.1",
               startdate=startdate)

summary(wmod)
```

```
Model fitted: Weibull (type 1) (4 parms)

Parameter estimates:

                 Estimate  Std. Error t-value   p-value    
b:(Intercept)  -4.5359008   0.6153678 -7.3710 7.834e-05 ***
c:(Intercept)   0.0008652   0.0079779  0.1084    0.9163    
d:(Intercept)   1.0204932   0.0765541 13.3304 9.585e-07 ***
e:(Intercept) 109.5974668   2.1918686 50.0018 2.834e-11 ***
---
Signif. codes:  0 '***' 0.001 '**' 0.01 '*' 0.05 '.' 0.1 ' ' 1

Residual standard error:

 0.01927989 (8 degrees of freedom)
```

```
plot(wmod)
```

```
AIC(wmod)
```

```
[1] -55.57968
```

#### GAM

```
Region<- "3"
```

```
gmod <- ndom_gam(r=Region,
               variant_int="JN.1",
               startdate=startdate)

summary(gmod)
```

```
Family: gaussian 
Link function: identity 

Formula:
share ~ s(t)

Parametric coefficients:
            Estimate Std. Error t value Pr(>|t|)    
(Intercept) 0.237534   0.001602   148.3 1.09e-06 ***
---
Signif. codes:  0 '***' 0.001 '**' 0.01 '*' 0.05 '.' 0.1 ' ' 1

Approximate significance of smooth terms:
       edf Ref.df    F  p-value    
s(t) 8.117  8.793 4114 1.32e-05 ***
---
Signif. codes:  0 '***' 0.001 '**' 0.01 '*' 0.05 '.' 0.1 ' ' 1

R-sq.(adj) =      1   Deviance explained =  100%
GCV = 0.00012815  Scale est. = 3.0791e-05  n = 12
```

```
plot(gmod)
```

```
AIC(gmod)
```

```
[1] -87.48317
```

```
rootfinder(mod = gmod,Range=c(1,150))
```

```
$root
[1] 118.4607

$f.root
[1] -6.698248e-09

$iter
[1] 7

$init.it
[1] NA

$estim.prec
[1] 6.103516e-05
```

#### Compile

```
ttd(modl=lmod,
    modw=wmod,
    modg=gmod,
    Region=Region,
    variant = "JN.1")->ttdj3
```

#### MSE

```
Variant<- "JN.1"
methodsu<-data.frame(Variant=Variant,
                     Region=Region,
                     t=gmod$model$t,
                    share=gmod$model$share,
                    y=gmod$fitted.values,
                    logistic=c(lmod$predres[,1]),
                    weibull=c(wmod$predres[,1])
                    ) 

MSE_JN1<- bind_rows(MSE_JN1,methodsu)
```

### Region 4

#### logistic

```
lmod <- ndom_l(r="4",
               variant_int="JN.1",
               startdate=startdate)

summary(lmod)
```

```
Model fitted: Generalised logistic (ED50 as parameter) (5 parms)

Parameter estimates:

                 Estimate  Std. Error t-value   p-value    
b:(Intercept) -9.0001e-02  1.8955e-02 -4.7481  0.002088 ** 
c:(Intercept)  3.6651e-05  8.7655e-03  0.0042  0.996780    
d:(Intercept)  8.1298e-01  2.3696e-02 34.3087 4.634e-09 ***
e:(Intercept)  1.1049e+02  8.3102e+00 13.2952 3.187e-06 ***
f:(Intercept)  1.0303e+00  5.5764e-01  1.8476  0.107140    
---
Signif. codes:  0 '***' 0.001 '**' 0.01 '*' 0.05 '.' 0.1 ' ' 1

Residual standard error:

 0.0176592 (7 degrees of freedom)
```

```
plot(lmod)
```

```
AIC(lmod)
```

```
[1] -57.28939
```

#### Weibull

```
wmod <- ndom_w(r="4",
               variant_int="JN.1",
               startdate=startdate)

summary(wmod)
```

```
Model fitted: Weibull (type 1) (4 parms)

Parameter estimates:

                 Estimate  Std. Error t-value   p-value    
b:(Intercept)  -5.7537697   0.8637765 -6.6612  0.000159 ***
c:(Intercept)   0.0010329   0.0095667  0.1080  0.916679    
d:(Intercept)   0.9090332   0.0559288 16.2534 2.065e-07 ***
e:(Intercept) 105.7250353   1.5711071 67.2933 2.647e-12 ***
---
Signif. codes:  0 '***' 0.001 '**' 0.01 '*' 0.05 '.' 0.1 ' ' 1

Residual standard error:

 0.02369242 (8 degrees of freedom)
```

```
plot(wmod)
```

```
AIC(wmod)
```

```
[1] -50.63346
```

#### GAM

```
Region<- "4"
```

```
gmod <- ndom_gam(r=Region,
               variant_int="JN.1",
               startdate=startdate)

summary(gmod)
```

```
Family: gaussian 
Link function: identity 

Formula:
share ~ s(t)

Parametric coefficients:
            Estimate Std. Error t value Pr(>|t|)    
(Intercept) 0.243676   0.004691   51.95 4.67e-06 ***
---
Signif. codes:  0 '***' 0.001 '**' 0.01 '*' 0.05 '.' 0.1 ' ' 1

Approximate significance of smooth terms:
       edf Ref.df     F  p-value    
s(t) 7.596   8.51 506.1 0.000132 ***
---
Signif. codes:  0 '***' 0.001 '**' 0.01 '*' 0.05 '.' 0.1 ' ' 1

R-sq.(adj) =  0.997   Deviance explained = 99.9%
GCV = 0.00093078  Scale est. = 0.00026401  n = 12
```

```
plot(gmod)
```

```
AIC(gmod)
```

```
[1] -60.74733
```

```
rootfinder(mod = gmod,Range=c(1,150))
```

```
$root
[1] 116.427

$f.root
[1] -3.441603e-07

$iter
[1] 7

$init.it
[1] NA

$estim.prec
[1] 6.103516e-05
```

#### Compile

```
ttd(modl=lmod,
    modw=wmod,
    modg=gmod,
    Region=Region,
    variant = "JN.1")->ttdj4
```

#### MSE

```
Variant<- "JN.1"
methodsu<-data.frame(Variant=Variant,
                     Region=Region,
                     t=gmod$model$t,
                    share=gmod$model$share,
                    y=gmod$fitted.values,
                    logistic=c(lmod$predres[,1]),
                    weibull=c(wmod$predres[,1])
                    ) 

MSE_JN1<- bind_rows(MSE_JN1,methodsu)
```

### Region 5

#### logistic

```
lmod <- ndom_l(r="5",
               variant_int="JN.1",
               startdate=startdate)

summary(lmod)
```

```
Model fitted: Generalised logistic (ED50 as parameter) (5 parms)

Parameter estimates:

                 Estimate  Std. Error t-value   p-value    
b:(Intercept)  -0.0558514   0.0090624 -6.1629 0.0004617 ***
c:(Intercept)  -0.0008071   0.0041077 -0.1965 0.8498109    
d:(Intercept)   0.8858222   0.0394216 22.4705 8.748e-08 ***
e:(Intercept) 111.3637494   9.3307233 11.9352 6.594e-06 ***
f:(Intercept)   1.5074840   0.6095245  2.4732 0.0426318 *  
---
Signif. codes:  0 '***' 0.001 '**' 0.01 '*' 0.05 '.' 0.1 ' ' 1

Residual standard error:

 0.00794512 (7 degrees of freedom)
```

```
plot(lmod)
```

```
AIC(lmod)
```

```
[1] -76.45817
```

#### Weibull

```
wmod <- ndom_w(r="5",
               variant_int="JN.1",
               startdate=startdate)

summary(wmod)
```

```
Model fitted: Weibull (type 1) (4 parms)

Parameter estimates:

                 Estimate  Std. Error t-value   p-value    
b:(Intercept)  -2.8917495   0.4178397 -6.9207 0.0001220 ***
c:(Intercept)   0.0002545   0.0060266  0.0422 0.9673513    
d:(Intercept)   1.3913698   0.2012076  6.9151 0.0001226 ***
e:(Intercept) 126.8494345   6.8495512 18.5194  7.45e-08 ***
---
Signif. codes:  0 '***' 0.001 '**' 0.01 '*' 0.05 '.' 0.1 ' ' 1

Residual standard error:

 0.014015 (8 degrees of freedom)
```

```
plot(wmod)
```

```
AIC(wmod)
```

```
[1] -63.23411
```

#### GAM

```
Region<- "5"
```

```
gmod <- ndom_gam(r=Region,
               variant_int="JN.1",
               startdate=startdate)

summary(gmod)
```

```
Family: gaussian 
Link function: identity 

Formula:
share ~ s(t)

Parametric coefficients:
            Estimate Std. Error t value Pr(>|t|)    
(Intercept) 0.210717   0.003242      65  5.5e-08 ***
---
Signif. codes:  0 '***' 0.001 '**' 0.01 '*' 0.05 '.' 0.1 ' ' 1

Approximate significance of smooth terms:
       edf Ref.df     F p-value    
s(t) 6.407  7.559 953.9  <2e-16 ***
---
Signif. codes:  0 '***' 0.001 '**' 0.01 '*' 0.05 '.' 0.1 ' ' 1

R-sq.(adj) =  0.998   Deviance explained = 99.9%
GCV = 0.0003295  Scale est. = 0.00012611  n = 12
```

```
plot(gmod)
```

```
AIC(gmod)
```

```
[1] -68.39578
```

```
rootfinder(mod = gmod,Range=c(1,150))
```

```
$root
[1] 125.6269

$f.root
[1] 1.363257e-07

$iter
[1] 6

$init.it
[1] NA

$estim.prec
[1] 6.103516e-05
```

#### Compile

```
ttd(modl=lmod,
    modw=wmod,
    modg=gmod,
    Region=Region,
    variant = "JN.1")->ttdj5
```

#### MSE

```
Variant<- "JN.1"
methodsu<-data.frame(Variant=Variant,
                     Region=Region,
                     t=gmod$model$t,
                    share=gmod$model$share,
                    y=gmod$fitted.values,
                    logistic=c(lmod$predres[,1]),
                    weibull=c(wmod$predres[,1])
                    ) 
MSE_JN1<- bind_rows(MSE_JN1,methodsu)
```

### Region 6

#### logistic

```
lmod <- ndom_l(r="6",
               variant_int="JN.1",
               startdate=startdate)

summary(lmod)
```

```
Model fitted: Generalised logistic (ED50 as parameter) (5 parms)

Parameter estimates:

                 Estimate  Std. Error  t-value   p-value    
b:(Intercept) -0.04879502  0.00433365 -11.2596 3.478e-06 ***
c:(Intercept) -0.00057964  0.00465287  -0.1246  0.903932    
d:(Intercept)  0.95474674  0.02036689  46.8774 4.741e-11 ***
e:(Intercept) 79.41983760 18.20331443   4.3629  0.002403 ** 
f:(Intercept)  5.09618276  3.58748785   1.4205  0.193229    
---
Signif. codes:  0 '***' 0.001 '**' 0.01 '*' 0.05 '.' 0.1 ' ' 1

Residual standard error:

 0.01074418 (8 degrees of freedom)
```

```
plot(lmod)
```

```
AIC(lmod)
```

```
[1] -75.28737
```

#### Weibull

```
wmod <- ndom_w(r="6",
               variant_int="JN.1",
               startdate=startdate)

summary(wmod)
```

```
Model fitted: Weibull (type 1) (4 parms)

Parameter estimates:

                 Estimate  Std. Error  t-value   p-value    
b:(Intercept) -4.3066e+00  3.0155e-01 -14.2813 1.727e-07 ***
c:(Intercept) -8.2542e-04  5.0370e-03  -0.1639    0.8735    
d:(Intercept)  1.0823e+00  4.0057e-02  27.0192 6.310e-10 ***
e:(Intercept)  1.1302e+02  1.2285e+00  92.0032 1.070e-14 ***
---
Signif. codes:  0 '***' 0.001 '**' 0.01 '*' 0.05 '.' 0.1 ' ' 1

Residual standard error:

 0.01243777 (9 degrees of freedom)
```

```
plot(wmod)
```

```
AIC(wmod)
```

```
[1] -71.95048
```

#### GAM

```
Region<- "6"
```

```
gmod <- ndom_gam(r=Region,
               variant_int="JN.1",
               startdate=startdate)

summary(gmod)
```

```
Family: gaussian 
Link function: identity 

Formula:
share ~ s(t)

Parametric coefficients:
            Estimate Std. Error t value Pr(>|t|)    
(Intercept) 0.280153   0.003999   70.05 3.11e-09 ***
---
Signif. codes:  0 '***' 0.001 '**' 0.01 '*' 0.05 '.' 0.1 ' ' 1

Approximate significance of smooth terms:
       edf Ref.df     F p-value    
s(t) 6.573  7.708 948.4  <2e-16 ***
---
Signif. codes:  0 '***' 0.001 '**' 0.01 '*' 0.05 '.' 0.1 ' ' 1

R-sq.(adj) =  0.998   Deviance explained = 99.9%
GCV = 0.00049807  Scale est. = 0.00020791  n = 13
```

```
plot(gmod)
```

```
AIC(gmod)
```

```
[1] -67.53762
```

```
rootfinder(mod = gmod,Range=c(1,150))
```

```
$root
[1] 120.7801

$f.root
[1] -5.926037e-12

$iter
[1] 6

$init.it
[1] NA

$estim.prec
[1] 8.123815e-05
```

#### Compile

```
ttd(modl=lmod,
    modw=wmod,
    modg=gmod,
    Region=Region,
    variant = "JN.1")->ttdj6
```

#### MSE

```
Variant<- "JN.1"
methodsu<-data.frame(Variant=Variant,
                     Region=Region,
                     t=gmod$model$t,
                    share=gmod$model$share,
                    y=gmod$fitted.values,
                    logistic=c(lmod$predres[,1]),
                    weibull=c(wmod$predres[,1])
                    ) 

MSE_JN1<- bind_rows(MSE_JN1,methodsu)
```

### Region 7

#### logistic

```
lmod <- ndom_l(r="7",
               variant_int="JN.1",
               startdate=startdate)

summary(lmod)
```

```
Model fitted: Generalised logistic (ED50 as parameter) (5 parms)

Parameter estimates:

                 Estimate  Std. Error t-value   p-value    
b:(Intercept)  -0.1522990   0.0470414 -3.2376   0.01192 *  
c:(Intercept)  -0.0007904   0.0088480 -0.0893   0.93101    
d:(Intercept)   0.7463807   0.0132000 56.5440 1.062e-11 ***
e:(Intercept) 126.0833174   3.4511468 36.5337 3.454e-10 ***
f:(Intercept)   0.3541620   0.1545276  2.2919   0.05111 .  
---
Signif. codes:  0 '***' 0.001 '**' 0.01 '*' 0.05 '.' 0.1 ' ' 1

Residual standard error:

 0.01679826 (8 degrees of freedom)
```

```
plot(lmod)
```

```
AIC(lmod)
```

```
[1] -63.66768
```

#### Weibull

```
wmod <- ndom_w(r="7",
               variant_int="JN.1",
               startdate=startdate)

summary(wmod)
```

```
Model fitted: Weibull (type 1) (4 parms)

Parameter estimates:

                 Estimate  Std. Error t-value   p-value    
b:(Intercept)  -4.9878044   1.2859660 -3.8786  0.003739 ** 
c:(Intercept)   0.0031171   0.0157561  0.1978  0.847572    
d:(Intercept)   0.8765035   0.0840728 10.4255 2.527e-06 ***
e:(Intercept) 107.8987374   2.6204874 41.1751 1.465e-11 ***
---
Signif. codes:  0 '***' 0.001 '**' 0.01 '*' 0.05 '.' 0.1 ' ' 1

Residual standard error:

 0.03674772 (9 degrees of freedom)
```

```
plot(wmod)
```

```
AIC(wmod)
```

```
[1] -43.78368
```

#### GAM

```
Region<- "7"
```

```
gmod <- ndom_gam(r=Region,
               variant_int="JN.1",
               startdate=startdate)

summary(gmod)
```

```
Family: gaussian 
Link function: identity 

Formula:
share ~ s(t)

Parametric coefficients:
             Estimate Std. Error t value Pr(>|t|)    
(Intercept) 0.2614220  0.0009116   286.8 8.19e-08 ***
---
Signif. codes:  0 '***' 0.001 '**' 0.01 '*' 0.05 '.' 0.1 ' ' 1

Approximate significance of smooth terms:
       edf Ref.df     F p-value    
s(t) 8.972      9 12562  <2e-16 ***
---
Signif. codes:  0 '***' 0.001 '**' 0.01 '*' 0.05 '.' 0.1 ' ' 1

R-sq.(adj) =      1   Deviance explained =  100%
GCV = 4.6379e-05  Scale est. = 1.0802e-05  n = 13
```

```
plot(gmod)
```

```
AIC(gmod)
```

```
[1] -108.77
```

```
rootfinder(mod = gmod,Range=c(1,150))
```

```
$root
[1] 121.3271

$f.root
[1] -1.912443e-07

$iter
[1] 7

$init.it
[1] NA

$estim.prec
[1] 6.103516e-05
```

#### Compile

```
ttd(modl=lmod,
    modw=wmod,
    modg=gmod,
    Region=Region,
    variant = "JN.1")->ttdj7
```

#### MSE

```
Variant<- "JN.1"
methodsu<-data.frame(Variant=Variant,
                     Region=Region,
                     t=gmod$model$t,
                    share=gmod$model$share,
                    y=gmod$fitted.values,
                    logistic=c(lmod$predres[,1]),
                    weibull=c(wmod$predres[,1])
                    ) 

MSE_JN1<- bind_rows(MSE_JN1,methodsu)
```

### Region 8

#### logistic

```
lmod <- ndom_l(r="8",
               variant_int="JN.1",
               startdate=startdate)

summary(lmod)
```

```
Model fitted: Generalised logistic (ED50 as parameter) (5 parms)

Parameter estimates:

                 Estimate  Std. Error t-value   p-value    
b:(Intercept) -6.1874e-02  1.3533e-02 -4.5721 0.0025677 ** 
c:(Intercept)  1.6382e-05  7.3990e-03  0.0022 0.9982952    
d:(Intercept)  9.0163e-01  4.8398e-02 18.6292 3.187e-07 ***
e:(Intercept)  1.0698e+02  1.4804e+01  7.2259 0.0001735 ***
f:(Intercept)  1.7827e+00  1.2220e+00  1.4588 0.1879765    
---
Signif. codes:  0 '***' 0.001 '**' 0.01 '*' 0.05 '.' 0.1 ' ' 1

Residual standard error:

 0.01529553 (7 degrees of freedom)
```

```
plot(lmod)
```

```
AIC(lmod)
```

```
[1] -60.7381
```

#### Weibull

```
wmod <- ndom_w(r="8",
               variant_int="JN.1",
               startdate=startdate)

summary(wmod)
```

```
Model fitted: Weibull (type 1) (4 parms)

Parameter estimates:

                 Estimate  Std. Error t-value   p-value    
b:(Intercept) -4.1879e+00  7.1333e-01 -5.8709 0.0003738 ***
c:(Intercept)  5.5977e-04  7.8390e-03  0.0714 0.9448252    
d:(Intercept)  1.1302e+00  1.2944e-01  8.7308 2.315e-05 ***
e:(Intercept)  1.1630e+02  3.6491e+00 31.8712 1.023e-09 ***
---
Signif. codes:  0 '***' 0.001 '**' 0.01 '*' 0.05 '.' 0.1 ' ' 1

Residual standard error:

 0.01886619 (8 degrees of freedom)
```

```
plot(wmod)
```

```
AIC(wmod)
```

```
[1] -56.10027
```

#### GAM

```
Region<- "8"
```

```
gmod <- ndom_gam(r=Region,
               variant_int="JN.1",
               startdate=startdate)

summary(gmod)
```

```
Family: gaussian 
Link function: identity 

Formula:
share ~ s(t)

Parametric coefficients:
            Estimate Std. Error t value Pr(>|t|)    
(Intercept) 0.222685   0.004773   46.66 5.33e-07 ***
---
Signif. codes:  0 '***' 0.001 '**' 0.01 '*' 0.05 '.' 0.1 ' ' 1

Approximate significance of smooth terms:
       edf Ref.df     F  p-value    
s(t) 6.686  7.812 493.5 1.63e-05 ***
---
Signif. codes:  0 '***' 0.001 '**' 0.01 '*' 0.05 '.' 0.1 ' ' 1

R-sq.(adj) =  0.997   Deviance explained = 99.9%
GCV = 0.00076027  Scale est. = 0.00027335  n = 12
```

```
plot(gmod)
```

```
AIC(gmod)
```

```
[1] -59.30681
```

```
rootfinder(mod = gmod,Range=c(1,150))
```

```
$root
[1] 122.1227

$f.root
[1] -5.055023e-11

$iter
[1] 7

$init.it
[1] NA

$estim.prec
[1] 6.103516e-05
```

#### Compile

```
ttd(modl=lmod,
    modw=wmod,
    modg=gmod,
    Region=Region,
    variant = "JN.1")->ttdj8
```

#### MSE

```
Variant<- "JN.1"
methodsu<-data.frame(Variant=Variant,
                     Region=Region,
                     t=gmod$model$t,
                    share=gmod$model$share,
                    y=gmod$fitted.values,
                    logistic=c(lmod$predres[,1]),
                    weibull=c(wmod$predres[,1])
                    ) 

MSE_JN1<- bind_rows(MSE_JN1,methodsu)
```

### Region 9

#### logistic

```
lmod <- ndom_l(r="9",
               variant_int="JN.1",
               startdate=startdate)

summary(lmod)
```

```
Model fitted: Generalised logistic (ED50 as parameter) (5 parms)

Parameter estimates:

                 Estimate  Std. Error t-value   p-value    
b:(Intercept)  -0.0693098   0.0079997 -8.6641 5.459e-05 ***
c:(Intercept)  -0.0009259   0.0041170 -0.2249  0.828486    
d:(Intercept)   0.9197941   0.0197769 46.5084 5.555e-10 ***
e:(Intercept) 110.6829760   5.4583542 20.2777 1.778e-07 ***
f:(Intercept)   1.2017860   0.3415145  3.5190  0.009741 ** 
---
Signif. codes:  0 '***' 0.001 '**' 0.01 '*' 0.05 '.' 0.1 ' ' 1

Residual standard error:

 0.00799782 (7 degrees of freedom)
```

```
plot(lmod)
```

```
AIC(lmod)
```

```
[1] -76.2995
```

#### Weibull

```
wmod <- ndom_w(r="9",
               variant_int="JN.1",
               startdate=startdate)

summary(wmod)
```

```
Model fitted: Weibull (type 1) (4 parms)

Parameter estimates:

                 Estimate  Std. Error t-value   p-value    
b:(Intercept) -3.8015e+00  5.0713e-01 -7.4961 6.953e-05 ***
c:(Intercept)  4.9436e-04  7.9750e-03  0.0620    0.9521    
d:(Intercept)  1.1912e+00  1.0650e-01 11.1853 3.657e-06 ***
e:(Intercept)  1.1208e+02  3.0717e+00 36.4868 3.489e-10 ***
---
Signif. codes:  0 '***' 0.001 '**' 0.01 '*' 0.05 '.' 0.1 ' ' 1

Residual standard error:

 0.01888899 (8 degrees of freedom)
```

```
plot(wmod)
```

```
AIC(wmod)
```

```
[1] -56.07129
```

#### GAM

```
Region<- "9"
```

```
gmod <- ndom_gam(r=Region,
               variant_int="JN.1",
               startdate=startdate)

summary(gmod)
```

```
Family: gaussian 
Link function: identity 

Formula:
share ~ s(t)

Parametric coefficients:
            Estimate Std. Error t value Pr(>|t|)    
(Intercept) 0.254366   0.001914   132.9 1.95e-06 ***
---
Signif. codes:  0 '***' 0.001 '**' 0.01 '*' 0.05 '.' 0.1 ' ' 1

Approximate significance of smooth terms:
       edf Ref.df    F  p-value    
s(t) 8.183  8.821 3220 1.68e-05 ***
---
Signif. codes:  0 '***' 0.001 '**' 0.01 '*' 0.05 '.' 0.1 ' ' 1

R-sq.(adj) =      1   Deviance explained =  100%
GCV = 0.00018723  Scale est. = 4.395e-05  n = 12
```

```
plot(gmod)
```

```
AIC(gmod)
```

```
[1] -83.3602
```

```
rootfinder(mod = gmod,Range=c(1,150))
```

```
$root
[1] 117.0712

$f.root
[1] 1.366462e-07

$iter
[1] 6

$init.it
[1] NA

$estim.prec
[1] 6.103516e-05
```

#### Compile

```
ttd(modl=lmod,
    modw=wmod,
    modg=gmod,
    Region=Region,
    variant = "JN.1")->ttdj9
```

#### MSE

```
Variant<- "JN.1"
methodsu<-data.frame(Variant=Variant,
                     Region=Region,
                     t=gmod$model$t,
                    share=gmod$model$share,
                    y=gmod$fitted.values,
                    logistic=c(lmod$predres[,1]),
                    weibull=c(wmod$predres[,1])
                    ) 

MSE_JN1<- bind_rows(MSE_JN1,methodsu)
```

### Region 10

#### logistic

```
lmod <- ndom_l(r="10",
               variant_int="JN.1",
               startdate=startdate)

summary(lmod)
```

```
Model fitted: Generalised logistic (ED50 as parameter) (5 parms)

Parameter estimates:

                 Estimate  Std. Error t-value   p-value    
b:(Intercept) -8.0215e-02  1.3655e-02 -5.8746 0.0001563 ***
c:(Intercept) -9.1912e-04  8.9337e-03 -0.1029 0.9200899    
d:(Intercept)  8.8050e-01  1.3117e-02 67.1269 1.311e-14 ***
e:(Intercept)  1.1680e+02  7.3612e+00 15.8665 2.036e-08 ***
f:(Intercept)  9.7225e-01  4.0601e-01  2.3946 0.0376590 *  
---
Signif. codes:  0 '***' 0.001 '**' 0.01 '*' 0.05 '.' 0.1 ' ' 1

Residual standard error:

 0.0184815 (10 degrees of freedom)
```

```
plot(lmod)
```

```
AIC(lmod)
```

```
[1] -71.24337
```

#### Weibull

```
wmod <- ndom_w(r="10",
               variant_int="JN.1",
               startdate=startdate)

summary(wmod)
```

```
Model fitted: Weibull (type 1) (4 parms)

Parameter estimates:

                 Estimate  Std. Error t-value   p-value    
b:(Intercept)  -5.4755165   0.7057772 -7.7581 8.736e-06 ***
c:(Intercept)   0.0027447   0.0118560  0.2315    0.8212    
d:(Intercept)   0.9449829   0.0326694 28.9257 9.911e-12 ***
e:(Intercept) 109.0586893   1.4336813 76.0690 2.371e-16 ***
---
Signif. codes:  0 '***' 0.001 '**' 0.01 '*' 0.05 '.' 0.1 ' ' 1

Residual standard error:

 0.02932914 (11 degrees of freedom)
```

```
plot(wmod)
```

```
AIC(wmod)
```

```
[1] -57.95938
```

#### GAM

```
Region<- "10"
```

```
gmod <- ndom_gam(r=Region,
               variant_int="JN.1",
               startdate=startdate)

summary(gmod)
```

```
Family: gaussian 
Link function: identity 

Formula:
share ~ s(t)

Parametric coefficients:
            Estimate Std. Error t value Pr(>|t|)    
(Intercept) 0.363045   0.003867   93.88 5.73e-11 ***
---
Signif. codes:  0 '***' 0.001 '**' 0.01 '*' 0.05 '.' 0.1 ' ' 1

Approximate significance of smooth terms:
       edf Ref.df    F p-value    
s(t) 7.832  8.646 1099  <2e-16 ***
---
Signif. codes:  0 '***' 0.001 '**' 0.01 '*' 0.05 '.' 0.1 ' ' 1

R-sq.(adj) =  0.999   Deviance explained = 99.9%
GCV = 0.00054551  Scale est. = 0.00022431  n = 15
```

```
plot(gmod)
```

```
AIC(gmod)
```

```
[1] -77.13515
```

```
rootfinder(mod = gmod,Range=c(1,150))
```

```
$root
[1] 120.0507

$f.root
[1] -2.021741e-07

$iter
[1] 6

$init.it
[1] NA

$estim.prec
[1] 6.103516e-05
```

#### Compile

```
ttd(modl=lmod,
    modw=wmod,
    modg=gmod,
    Region=Region,
    variant = "JN.1")->ttdj10
```

#### MSE

```
Variant<- "JN.1"
methodsu<-data.frame(Variant=Variant,
                     Region=Region,
                     t=gmod$model$t,
                    share=gmod$model$share,
                    y=gmod$fitted.values,
                    logistic=c(lmod$predres[,1]),
                    weibull=c(wmod$predres[,1])
                    ) 

MSE_JN1<- bind_rows(MSE_JN1,methodsu)
```

## XBB.1.5

```
startdate <- as.Date("2022-10-15")
```

### USA

#### logistic

```
lmod <- ndom_l(r="USA",
               variant_int="XBB.1.5",
               startdate=startdate)

summary(lmod)
```

```
Model fitted: Generalised logistic (ED50 as parameter) (5 parms)

Parameter estimates:

                 Estimate  Std. Error  t-value   p-value    
b:(Intercept)  -0.0714266   0.0031225 -22.8748 7.732e-08 ***
c:(Intercept)  -0.0012767   0.0021011  -0.6076    0.5626    
d:(Intercept)   0.8461975   0.0054656 154.8238 1.238e-13 ***
e:(Intercept) 106.0857695   1.7685445  59.9848 9.394e-11 ***
f:(Intercept)   0.8966647   0.0861382  10.4096 1.641e-05 ***
---
Signif. codes:  0 '***' 0.001 '**' 0.01 '*' 0.05 '.' 0.1 ' ' 1

Residual standard error:

 0.003328147 (7 degrees of freedom)
```

```
plot(lmod)
```

```
AIC(lmod)
```

```
[1] -97.34158
```

#### Weibull

```
wmod <- ndom_w(r="USA",
               variant_int="XBB.1.5",
               startdate=startdate)

summary(wmod)
```

```
Model fitted: Weibull (type 1) (4 parms)

Parameter estimates:

                 Estimate  Std. Error t-value   p-value    
b:(Intercept) -3.1206e+00  5.5474e-01 -5.6253 0.0004955 ***
c:(Intercept)  5.3375e-04  1.1422e-02  0.0467 0.9638735    
d:(Intercept)  1.1112e+00  1.2268e-01  9.0581 1.767e-05 ***
e:(Intercept)  1.0145e+02  4.0757e+00 24.8911 7.258e-09 ***
---
Signif. codes:  0 '***' 0.001 '**' 0.01 '*' 0.05 '.' 0.1 ' ' 1

Residual standard error:

 0.02429101 (8 degrees of freedom)
```

```
plot(wmod)
```

```
AIC(wmod)
```

```
[1] -50.03464
```

#### GAM

```
Region<- "USA"
```

```
gmod <- ndom_gam(r=Region,
               variant_int="XBB.1.5",
               startdate=startdate)

summary(gmod)
```

```
Family: gaussian 
Link function: identity 

Formula:
share ~ s(t)

Parametric coefficients:
             Estimate Std. Error t value Pr(>|t|)    
(Intercept) 0.2892422  0.0003326   869.6 4.85e-07 ***
---
Signif. codes:  0 '***' 0.001 '**' 0.01 '*' 0.05 '.' 0.1 ' ' 1

Approximate significance of smooth terms:
       edf Ref.df      F  p-value    
s(t) 8.835  8.992 101596 1.54e-05 ***
---
Signif. codes:  0 '***' 0.001 '**' 0.01 '*' 0.05 '.' 0.1 ' ' 1

R-sq.(adj) =      1   Deviance explained =  100%
GCV = 7.3587e-06  Scale est. = 1.3275e-06  n = 12
```

```
plot(gmod)
```

```
AIC(gmod)
```

```
[1] -127.2121
```

```
rootfinder(mod = gmod,Range=c(1,150))
```

```
$root
[1] 109.4205

$f.root
[1] 1.158e-07

$iter
[1] 6

$init.it
[1] NA

$estim.prec
[1] 6.103516e-05
```

#### Compile

```
ttd(modl=lmod,
    modw=wmod,
    modg=gmod,
    Region=Region,
    variant = "XBB.1.5")->ttdxu
```

#### MSE

```
Variant<- "XBB.1.5"
methodsu<-data.frame(Variant=Variant,
                     Region=Region,
                     t=gmod$model$t,
                    share=gmod$model$share,
                    y=gmod$fitted.values,
                    logistic=c(lmod$predres[,1]),
                    weibull=c(wmod$predres[,1])
                    ) 

MSE_XBB<- bind_rows(MSE_XBB,methodsu)
```

### Region 1

#### logistic

```
lmod <- ndom_l(r="1",
               variant_int="XBB.1.5",
               startdate=startdate)

summary(lmod)
```

```
Model fitted: Generalised logistic (ED50 as parameter) (5 parms)

Parameter estimates:

                Estimate Std. Error t-value   p-value    
b:(Intercept) -0.0745070  0.0496942 -1.4993 0.1940757    
c:(Intercept) -0.0032412  0.0273280 -0.1186 0.9102071    
d:(Intercept)  0.9590140  0.1108479  8.6516 0.0003409 ***
e:(Intercept) 92.3320380 19.2386194  4.7993 0.0048866 ** 
f:(Intercept)  0.7707986  0.9482112  0.8129 0.4532290    
---
Signif. codes:  0 '***' 0.001 '**' 0.01 '*' 0.05 '.' 0.1 ' ' 1

Residual standard error:

 0.03094922 (5 degrees of freedom)
```

```
plot(lmod)
```

```
AIC(lmod)
```

```
[1] -36.06085
```

#### Weibull

```
wmod <- ndom_w(r="1",
               variant_int="XBB.1.5",
               startdate=startdate)

summary(wmod)
```

```
Model fitted: Weibull (type 1) (4 parms)

Parameter estimates:

                 Estimate  Std. Error t-value  p-value   
b:(Intercept)  -1.7279694   0.6085595 -2.8394 0.029589 * 
c:(Intercept)   0.0023734   0.0205532  0.1155 0.911835   
d:(Intercept)   1.9082535   0.7756919  2.4601 0.049115 * 
e:(Intercept) 104.4928025  25.3226633  4.1265 0.006171 **
---
Signif. codes:  0 '***' 0.001 '**' 0.01 '*' 0.05 '.' 0.1 ' ' 1

Residual standard error:

 0.03671747 (6 degrees of freedom)
```

```
plot(wmod)
```

```
AIC(wmod)
```

```
[1] -32.81954
```

#### GAM

```
Region<- "1"
```

```
gmod <- ndom_gam(r=Region,
               variant_int="XBB.1.5",
               startdate=startdate)

summary(gmod)
```

```
Family: gaussian 
Link function: identity 

Formula:
share ~ s(t)

Parametric coefficients:
            Estimate Std. Error t value Pr(>|t|)    
(Intercept)  0.31909    0.00989   32.27 2.92e-06 ***
---
Signif. codes:  0 '***' 0.001 '**' 0.01 '*' 0.05 '.' 0.1 ' ' 1

Approximate significance of smooth terms:
       edf Ref.df     F  p-value    
s(t) 4.733  5.807 198.9 7.37e-05 ***
---
Signif. codes:  0 '***' 0.001 '**' 0.01 '*' 0.05 '.' 0.1 ' ' 1

R-sq.(adj) =  0.992   Deviance explained = 99.6%
GCV = 0.002292  Scale est. = 0.00097803  n = 10
```

```
plot(gmod)
```

```
AIC(gmod)
```

```
[1] -35.97156
```

```
rootfinder(mod = gmod,Range=c(1,150))
```

```
$root
[1] 89.02569

$f.root
[1] -4.618947e-10

$iter
[1] 7

$init.it
[1] NA

$estim.prec
[1] 6.103516e-05
```

#### Compile

```
ttd(modl=lmod,
    modw=wmod,
    modg=gmod,
    Region=Region,
    variant = "XBB.1.5")->ttdx1
```

#### MSE

```
Variant<- "XBB.1.5"
methodsu<-data.frame(Variant=Variant,
                     Region=Region,
                     t=gmod$model$t,
                    share=gmod$model$share,
                    y=gmod$fitted.values,
                    logistic=c(lmod$predres[,1]),
                    weibull=c(wmod$predres[,1])
                    ) 

MSE_XBB<- bind_rows(MSE_XBB,methodsu)
```

### Region 2

#### logistic

```
lmod <- ndom_l(r="2",
               variant_int="XBB.1.5",
               startdate=startdate)

summary(lmod)
```

```
Model fitted: Generalised logistic (ED50 as parameter) (5 parms)

Parameter estimates:

                Estimate Std. Error  t-value  p-value    
b:(Intercept) -0.0525027  0.0041432 -12.6720 1.48e-05 ***
c:(Intercept) -0.0010455  0.0061657  -0.1696  0.87092    
d:(Intercept)  0.9286513  0.0120101  77.3227 3.15e-10 ***
e:(Intercept) 46.6774646 13.9108804   3.3555  0.01532 *  
f:(Intercept)  3.7704758  2.1454598   1.7574  0.12936    
---
Signif. codes:  0 '***' 0.001 '**' 0.01 '*' 0.05 '.' 0.1 ' ' 1

Residual standard error:

 0.00933201 (6 degrees of freedom)
```

```
plot(lmod)
```

```
AIC(lmod)
```

```
[1] -66.28555
```

#### Weibull

```
wmod <- ndom_w(r="2",
               variant_int="XBB.1.5",
               startdate=startdate)

summary(wmod)
```

```
Model fitted: Weibull (type 1) (4 parms)

Parameter estimates:

                Estimate Std. Error t-value   p-value    
b:(Intercept) -2.8711974  0.3024824 -9.4921 3.013e-05 ***
c:(Intercept)  0.0004438  0.0104814  0.0423    0.9674    
d:(Intercept)  1.0618462  0.0501300 21.1819 1.316e-07 ***
e:(Intercept) 71.2682174  1.4933450 47.7239 4.640e-10 ***
---
Signif. codes:  0 '***' 0.001 '**' 0.01 '*' 0.05 '.' 0.1 ' ' 1

Residual standard error:

 0.01927414 (7 degrees of freedom)
```

```
plot(wmod)
```

```
AIC(wmod)
```

```
[1] -50.63298
```

#### GAM

```
Region<- "2"
```

```
gmod <- ndom_gam(r=Region,
               variant_int="XBB.1.5",
               startdate=startdate)

summary(gmod)
```

```
Family: gaussian 
Link function: identity 

Formula:
share ~ s(t)

Parametric coefficients:
            Estimate Std. Error t value Pr(>|t|)    
(Intercept) 0.403744   0.001101   366.8 0.000286 ***
---
Signif. codes:  0 '***' 0.001 '**' 0.01 '*' 0.05 '.' 0.1 ' ' 1

Approximate significance of smooth terms:
       edf Ref.df     F p-value   
s(t) 8.679  8.971 12222 0.00702 **
---
Signif. codes:  0 '***' 0.001 '**' 0.01 '*' 0.05 '.' 0.1 ' ' 1

R-sq.(adj) =      1   Deviance explained =  100%
GCV = 0.00011095  Scale est. = 1.3326e-05  n = 11
```

```
plot(gmod)
```

```
AIC(gmod)
```

```
[1] -94.22289
```

```
rootfinder(mod = gmod,Range=c(1,150))
```

```
$root
[1] 79.77624

$f.root
[1] -9.856322e-09

$iter
[1] 5

$init.it
[1] NA

$estim.prec
[1] 6.103516e-05
```

#### Compile

```
ttd(modl=lmod,
    modw=wmod,
    modg=gmod,
    Region=Region,
    variant = "XBB.1.5")->ttdx2
```

#### MSE

```
Variant<- "XBB.1.5"
methodsu<-data.frame(Variant=Variant,
                     Region=Region,
                     t=gmod$model$t,
                    share=gmod$model$share,
                    y=gmod$fitted.values,
                    logistic=c(lmod$predres[,1]),
                    weibull=c(wmod$predres[,1])
                    ) 

MSE_XBB<- bind_rows(MSE_XBB,methodsu)
```

### Region 3

#### logistic

```
lmod <- ndom_l(r="3",
               variant_int="XBB.1.5",
               startdate=startdate)

summary(lmod)
```

```
Model fitted: Generalised logistic (ED50 as parameter) (5 parms)

Parameter estimates:

                Estimate Std. Error  t-value   p-value    
b:(Intercept) -0.0820047  0.0048533 -16.8968 6.229e-07 ***
c:(Intercept) -0.0010652  0.0034843  -0.3057 0.7687167    
d:(Intercept)  0.8771878  0.0054873 159.8577 9.897e-14 ***
e:(Intercept) 92.6912626  2.5986795  35.6686 3.535e-09 ***
f:(Intercept)  1.0093466  0.1537770   6.5637 0.0003148 ***
---
Signif. codes:  0 '***' 0.001 '**' 0.01 '*' 0.05 '.' 0.1 ' ' 1

Residual standard error:

 0.005848005 (7 degrees of freedom)
```

```
plot(lmod)
```

```
AIC(lmod)
```

```
[1] -83.81315
```

#### Weibull

```
wmod <- ndom_w(r="3",
               variant_int="XBB.1.5",
               startdate=startdate)

summary(wmod)
```

```
Model fitted: Weibull (type 1) (4 parms)

Parameter estimates:

                Estimate Std. Error t-value   p-value    
b:(Intercept) -4.2299443  0.5539139 -7.6365 6.094e-05 ***
c:(Intercept)  0.0040655  0.0123349  0.3296    0.7502    
d:(Intercept)  0.9794935  0.0463227 21.1450 2.629e-08 ***
e:(Intercept) 86.6505779  1.5147636 57.2040 9.682e-12 ***
---
Signif. codes:  0 '***' 0.001 '**' 0.01 '*' 0.05 '.' 0.1 ' ' 1

Residual standard error:

 0.02663116 (8 degrees of freedom)
```

```
plot(wmod)
```

```
AIC(wmod)
```

```
[1] -47.82722
```

#### GAM

```
Region<- "3"
```

```
gmod <- ndom_gam(r=Region,
               variant_int="XBB.1.5",
               startdate=startdate)

summary(gmod)
```

```
Family: gaussian 
Link function: identity 

Formula:
share ~ s(t)

Parametric coefficients:
            Estimate Std. Error t value Pr(>|t|)    
(Intercept) 0.354615   0.001754   202.2 2.05e-07 ***
---
Signif. codes:  0 '***' 0.001 '**' 0.01 '*' 0.05 '.' 0.1 ' ' 1

Approximate significance of smooth terms:
       edf Ref.df    F p-value    
s(t) 7.939  8.708 4881 1.2e-05 ***
---
Signif. codes:  0 '***' 0.001 '**' 0.01 '*' 0.05 '.' 0.1 ' ' 1

R-sq.(adj) =      1   Deviance explained =  100%
GCV = 0.00014478  Scale est. = 3.6926e-05  n = 12
```

```
plot(gmod)
```

```
AIC(gmod)
```

```
[1] -84.94152
```

```
rootfinder(mod = gmod,Range=c(1,150))
```

```
$root
[1] 96.59323

$f.root
[1] 4.097823e-07

$iter
[1] 6

$init.it
[1] NA

$estim.prec
[1] 6.103516e-05
```

#### Compile

```
ttd(modl=lmod,
    modw=wmod,
    modg=gmod,
    Region=Region,
    variant = "XBB.1.5")->ttdx3
```

#### MSE

```
Variant<- "XBB.1.5"
methodsu<-data.frame(Variant=Variant,
                     Region=Region,
                     t=gmod$model$t,
                    share=gmod$model$share,
                    y=gmod$fitted.values,
                    logistic=c(lmod$predres[,1]),
                    weibull=c(wmod$predres[,1])
                    ) 

MSE_XBB<- bind_rows(MSE_XBB,methodsu)
```

### Region 4

#### logistic

```
lmod <- ndom_l(r="4",
               variant_int="XBB.1.5",
               startdate=startdate)

summary(lmod)
```

```
Model fitted: Generalised logistic (ED50 as parameter) (5 parms)

Parameter estimates:

                 Estimate  Std. Error t-value   p-value    
b:(Intercept)  -0.0752148   0.0090831 -8.2807 3.404e-05 ***
c:(Intercept)  -0.0013259   0.0063563 -0.2086  0.839971    
d:(Intercept)   0.8490541   0.0111713 76.0030 1.001e-12 ***
e:(Intercept) 106.7631269   5.4707240 19.5154 4.940e-08 ***
f:(Intercept)   0.9386070   0.2773797  3.3838  0.009587 ** 
---
Signif. codes:  0 '***' 0.001 '**' 0.01 '*' 0.05 '.' 0.1 ' ' 1

Residual standard error:

 0.0111411 (8 degrees of freedom)
```

```
plot(lmod)
```

```
AIC(lmod)
```

```
[1] -74.34416
```

#### Weibull

```
wmod <- ndom_w(r="4",
               variant_int="XBB.1.5",
               startdate=startdate)

summary(wmod)
```

```
Model fitted: Weibull (type 1) (4 parms)

Parameter estimates:

                Estimate Std. Error t-value   p-value    
b:(Intercept) -4.1622924  0.5982458 -6.9575 6.629e-05 ***
c:(Intercept)  0.0014786  0.0119120  0.1241    0.9039    
d:(Intercept)  0.9667199  0.0553453 17.4671 2.987e-08 ***
e:(Intercept) 98.9253736  1.8777655 52.6825 1.607e-12 ***
---
Signif. codes:  0 '***' 0.001 '**' 0.01 '*' 0.05 '.' 0.1 ' ' 1

Residual standard error:

 0.02699566 (9 degrees of freedom)
```

```
plot(wmod)
```

```
AIC(wmod)
```

```
[1] -51.80208
```

#### GAM

```
Region<- "4"
```

```
gmod <- ndom_gam(r=Region,
               variant_int="XBB.1.5",
               startdate=startdate)

summary(gmod)
```

```
Family: gaussian 
Link function: identity 

Formula:
share ~ s(t)

Parametric coefficients:
            Estimate Std. Error t value Pr(>|t|)    
(Intercept) 0.323487   0.001532   211.2 6.58e-09 ***
---
Signif. codes:  0 '***' 0.001 '**' 0.01 '*' 0.05 '.' 0.1 ' ' 1

Approximate significance of smooth terms:
       edf Ref.df    F p-value    
s(t) 8.182  8.819 5689  <2e-16 ***
---
Signif. codes:  0 '***' 0.001 '**' 0.01 '*' 0.05 '.' 0.1 ' ' 1

R-sq.(adj) =      1   Deviance explained =  100%
GCV = 0.00010385  Scale est. = 3.0501e-05  n = 13
```

```
plot(gmod)
```

```
AIC(gmod)
```

```
[1] -93.84207
```

```
rootfinder(mod = gmod,Range=c(1,150))
```

```
$root
[1] 111.3224

$f.root
[1] -7.726466e-09

$iter
[1] 6

$init.it
[1] NA

$estim.prec
[1] 6.103516e-05
```

#### Compile

```
ttd(modl=lmod,
    modw=wmod,
    modg=gmod,
    Region=Region,
    variant = "XBB.1.5")->ttdx4
```

#### MSE

```
Variant<- "XBB.1.5"
methodsu<-data.frame(Variant=Variant,
                     Region=Region,
                     t=gmod$model$t,
                    share=gmod$model$share,
                    y=gmod$fitted.values,
                    logistic=c(lmod$predres[,1]),
                    weibull=c(wmod$predres[,1])
                    ) 

MSE_XBB<- bind_rows(MSE_XBB,methodsu)
```

### Region 5

#### logistic

```
lmod <- ndom_l(r="5",
               variant_int="XBB.1.5",
               startdate=startdate)

summary(lmod)
```

```
Model fitted: Generalised logistic (ED50 as parameter) (5 parms)

Parameter estimates:

                 Estimate  Std. Error t-value   p-value    
b:(Intercept) -7.5327e-02  8.8347e-03 -8.5262 6.055e-05 ***
c:(Intercept) -3.2748e-04  4.8237e-03 -0.0679   0.94777    
d:(Intercept)  8.6073e-01  1.6485e-02 52.2137 2.476e-10 ***
e:(Intercept)  1.0211e+02  6.5559e+00 15.5760 1.087e-06 ***
f:(Intercept)  1.5191e+00  5.4554e-01  2.7845   0.02712 *  
---
Signif. codes:  0 '***' 0.001 '**' 0.01 '*' 0.05 '.' 0.1 ' ' 1

Residual standard error:

 0.0101711 (7 degrees of freedom)
```

```
plot(lmod)
```

```
AIC(lmod)
```

```
[1] -70.53034
```

#### Weibull

```
wmod <- ndom_w(r="5",
               variant_int="XBB.1.5",
               startdate=startdate)

summary(wmod)
```

```
Model fitted: Weibull (type 1) (4 parms)

Parameter estimates:

                 Estimate  Std. Error t-value   p-value    
b:(Intercept) -4.9416e+00  6.0052e-01 -8.2289 3.562e-05 ***
c:(Intercept)  3.4942e-04  7.9381e-03  0.0440     0.966    
d:(Intercept)  9.8846e-01  5.4986e-02 17.9766 9.404e-08 ***
e:(Intercept)  1.0463e+02  1.5003e+00 69.7396 1.990e-12 ***
---
Signif. codes:  0 '***' 0.001 '**' 0.01 '*' 0.05 '.' 0.1 ' ' 1

Residual standard error:

 0.01902119 (8 degrees of freedom)
```

```
plot(wmod)
```

```
AIC(wmod)
```

```
[1] -55.9039
```

#### GAM

```
Region<- "5"
```

```
gmod <- ndom_gam(r=Region,
               variant_int="XBB.1.5",
               startdate=startdate)

summary(gmod)
```

```
Family: gaussian 
Link function: identity 

Formula:
share ~ s(t)

Parametric coefficients:
            Estimate Std. Error t value Pr(>|t|)    
(Intercept) 0.260039   0.004171   62.35 4.66e-07 ***
---
Signif. codes:  0 '***' 0.001 '**' 0.01 '*' 0.05 '.' 0.1 ' ' 1

Approximate significance of smooth terms:
       edf Ref.df     F  p-value    
s(t) 7.053  8.121 744.2 9.47e-06 ***
---
Signif. codes:  0 '***' 0.001 '**' 0.01 '*' 0.05 '.' 0.1 ' ' 1

R-sq.(adj) =  0.998   Deviance explained = 99.9%
GCV = 0.00063464  Scale est. = 0.00020873  n = 12
```

```
plot(gmod)
```

```
AIC(gmod)
```

```
[1] -62.87706
```

```
rootfinder(mod = gmod,Range=c(1,150))
```

```
$root
[1] 113.5244

$f.root
[1] -1.199357e-07

$iter
[1] 7

$init.it
[1] NA

$estim.prec
[1] 6.103516e-05
```

#### Compile

```
ttd(modl=lmod,
    modw=wmod,
    modg=gmod,
    Region=Region,
    variant = "XBB.1.5")->ttdx5
```

#### MSE

```
Variant<- "XBB.1.5"
methodsu<-data.frame(Variant=Variant,
                     Region=Region,
                     t=gmod$model$t,
                    share=gmod$model$share,
                    y=gmod$fitted.values,
                    logistic=c(lmod$predres[,1]),
                    weibull=c(wmod$predres[,1])
                    ) 

MSE_XBB<- bind_rows(MSE_XBB,methodsu)
```

### Region 6

#### logistic

```
lmod <- ndom_l(r="6",
               variant_int="XBB.1.5",
               startdate=startdate)

summary(lmod)
```

```
Model fitted: Generalised logistic (ED50 as parameter) (5 parms)

Parameter estimates:

                 Estimate  Std. Error  t-value   p-value    
b:(Intercept) -7.4959e-02  7.3171e-03 -10.2444 1.824e-05 ***
c:(Intercept) -8.6739e-04  3.6834e-03  -0.2355  0.820570    
d:(Intercept)  8.9773e-01  1.5376e-02  58.3855 1.135e-10 ***
e:(Intercept)  1.1353e+02  3.6545e+00  31.0640 9.251e-09 ***
f:(Intercept)  9.8422e-01  2.0755e-01   4.7421  0.002103 ** 
---
Signif. codes:  0 '***' 0.001 '**' 0.01 '*' 0.05 '.' 0.1 ' ' 1

Residual standard error:

 0.006965029 (7 degrees of freedom)
```

```
plot(lmod)
```

```
AIC(lmod)
```

```
[1] -79.61792
```

#### Weibull

```
wmod <- ndom_w(r="6",
               variant_int="XBB.1.5",
               startdate=startdate)

summary(wmod)
```

```
Model fitted: Weibull (type 1) (4 parms)

Parameter estimates:

                 Estimate  Std. Error t-value   p-value    
b:(Intercept) -3.6217e+00  5.7755e-01 -6.2708 0.0002402 ***
c:(Intercept)  3.4702e-04  9.2017e-03  0.0377 0.9708413    
d:(Intercept)  1.2035e+00  1.3119e-01  9.1740 1.609e-05 ***
e:(Intercept)  1.1199e+02  3.8606e+00 29.0093 2.158e-09 ***
---
Signif. codes:  0 '***' 0.001 '**' 0.01 '*' 0.05 '.' 0.1 ' ' 1

Residual standard error:

 0.02132975 (8 degrees of freedom)
```

```
plot(wmod)
```

```
AIC(wmod)
```

```
[1] -53.15472
```

#### GAM

```
Region<- "6"
```

```
gmod <- ndom_gam(r=Region,
               variant_int="XBB.1.5",
               startdate=startdate)

summary(gmod)
```

```
Family: gaussian 
Link function: identity 

Formula:
share ~ s(t)

Parametric coefficients:
            Estimate Std. Error t value Pr(>|t|)    
(Intercept) 0.256618   0.001571   163.4 4.52e-06 ***
---
Signif. codes:  0 '***' 0.001 '**' 0.01 '*' 0.05 '.' 0.1 ' ' 1

Approximate significance of smooth terms:
       edf Ref.df    F  p-value    
s(t) 8.516  8.935 4649 0.000213 ***
---
Signif. codes:  0 '***' 0.001 '**' 0.01 '*' 0.05 '.' 0.1 ' ' 1

R-sq.(adj) =      1   Deviance explained =  100%
GCV = 0.00014299  Scale est. = 2.9599e-05  n = 12
```

```
plot(gmod)
```

```
AIC(gmod)
```

```
[1] -88.94707
```

```
rootfinder(mod = gmod,Range=c(1,150))
```

```
$root
[1] 116.27

$f.root
[1] -3.129574e-10

$iter
[1] 7

$init.it
[1] NA

$estim.prec
[1] 6.103516e-05
```

#### Compile

```
ttd(modl=lmod,
    modw=wmod,
    modg=gmod,
    Region=Region,
    variant = "XBB.1.5")->ttdx6
```

#### MSE

```
Variant<- "XBB.1.5"
methodsu<-data.frame(Variant=Variant,
                     Region=Region,
                     t=gmod$model$t,
                    share=gmod$model$share,
                    y=gmod$fitted.values,
                    logistic=c(lmod$predres[,1]),
                    weibull=c(wmod$predres[,1])
                    ) 

MSE_XBB<- bind_rows(MSE_XBB,methodsu)
```

### Region 7

#### logistic

```
lmod <- ndom_l(r="7",
               variant_int="XBB.1.5",
               startdate=startdate)

summary(lmod)
```

```
Model fitted: Generalised logistic (ED50 as parameter) (5 parms)

Parameter estimates:

                 Estimate  Std. Error t-value   p-value    
b:(Intercept) -1.0155e-01  1.9232e-02 -5.2800 0.0005071 ***
c:(Intercept) -8.6456e-04  8.0332e-03 -0.1076 0.9166560    
d:(Intercept)  8.5367e-01  1.2150e-02 70.2611 1.212e-13 ***
e:(Intercept)  1.1337e+02  5.9376e+00 19.0932 1.367e-08 ***
f:(Intercept)  8.6737e-01  3.6982e-01  2.3454 0.0436339 *  
---
Signif. codes:  0 '***' 0.001 '**' 0.01 '*' 0.05 '.' 0.1 ' ' 1

Residual standard error:

 0.01703579 (9 degrees of freedom)
```

```
plot(lmod)
```

```
AIC(lmod)
```

```
[1] -68.48366
```

#### Weibull

```
wmod <- ndom_w(r="7",
               variant_int="XBB.1.5",
               startdate=startdate)

summary(wmod)
```

```
Model fitted: Weibull (type 1) (4 parms)

Parameter estimates:

                 Estimate  Std. Error t-value   p-value    
b:(Intercept) -6.5925e+00  8.9405e-01 -7.3738 2.387e-05 ***
c:(Intercept)  4.3745e-04  1.1119e-02  0.0393    0.9694    
d:(Intercept)  9.0290e-01  2.8431e-02 31.7578 2.254e-11 ***
e:(Intercept)  1.0507e+02  1.2111e+00 86.7518 9.507e-16 ***
---
Signif. codes:  0 '***' 0.001 '**' 0.01 '*' 0.05 '.' 0.1 ' ' 1

Residual standard error:

 0.02766609 (10 degrees of freedom)
```

```
plot(wmod)
```

```
AIC(wmod)
```

```
[1] -55.43167
```

#### GAM

```
Region<- "7"
```

```
gmod <- ndom_gam(r=Region,
               variant_int="XBB.1.5",
               startdate=startdate)

summary(gmod)
```

```
Family: gaussian 
Link function: identity 

Formula:
share ~ s(t)

Parametric coefficients:
            Estimate Std. Error t value Pr(>|t|)    
(Intercept)  0.33966    0.00426   79.73 1.17e-08 ***
---
Signif. codes:  0 '***' 0.001 '**' 0.01 '*' 0.05 '.' 0.1 ' ' 1

Approximate significance of smooth terms:
       edf Ref.df   F p-value    
s(t) 8.218  8.833 848  <2e-16 ***
---
Signif. codes:  0 '***' 0.001 '**' 0.01 '*' 0.05 '.' 0.1 ' ' 1

R-sq.(adj) =  0.998   Deviance explained = 99.9%
GCV = 0.00074382  Scale est. = 0.00025409  n = 14
```

```
plot(gmod)
```

```
AIC(gmod)
```

```
[1] -70.7618
```

```
rootfinder(mod = gmod,Range=c(1,150))
```

```
$root
[1] 114.894

$f.root
[1] -1.720382e-07

$iter
[1] 7

$init.it
[1] NA

$estim.prec
[1] 6.103516e-05
```

#### Compile

```
ttd(modl=lmod,
    modw=wmod,
    modg=gmod,
    Region=Region,
    variant = "XBB.1.5")->ttdx7
```

#### MSE

```
Variant<- "XBB.1.5"
methodsu<-data.frame(Variant=Variant,
                     Region=Region,
                     t=gmod$model$t,
                    share=gmod$model$share,
                    y=gmod$fitted.values,
                    logistic=c(lmod$predres[,1]),
                    weibull=c(wmod$predres[,1])
                    ) 

MSE_XBB<- bind_rows(MSE_XBB,methodsu)
```

### Region 8

#### logistic

```
lmod <- ndom_l(r="8",
               variant_int="XBB.1.5",
               startdate=startdate)

summary(lmod)
```

```
Model fitted: Generalised logistic (ED50 as parameter) (5 parms)

Parameter estimates:

                 Estimate  Std. Error  t-value   p-value    
b:(Intercept) -6.5579e-02  6.5193e-03 -10.0591 2.058e-05 ***
c:(Intercept)  4.3441e-05  5.3644e-03   0.0081  0.993765    
d:(Intercept)  8.8827e-01  1.6328e-02  54.4030 1.859e-10 ***
e:(Intercept)  7.6959e+01  1.6245e+01   4.7375  0.002114 ** 
f:(Intercept)  4.7684e+00  4.0471e+00   1.1782  0.277195    
---
Signif. codes:  0 '***' 0.001 '**' 0.01 '*' 0.05 '.' 0.1 ' ' 1

Residual standard error:

 0.0121766 (7 degrees of freedom)
```

```
plot(lmod)
```

```
AIC(lmod)
```

```
[1] -66.21118
```

#### Weibull

```
wmod <- ndom_w(r="8",
               variant_int="XBB.1.5",
               startdate=startdate)

summary(wmod)
```

```
Model fitted: Weibull (type 1) (4 parms)

Parameter estimates:

                Estimate Std. Error  t-value   p-value    
b:(Intercept) -5.6284274  0.3506031 -16.0536 2.274e-07 ***
c:(Intercept)  0.0010635  0.0049865   0.2133    0.8364    
d:(Intercept)  0.9493738  0.0225444  42.1113 1.114e-10 ***
e:(Intercept) 99.7206141  0.6734173 148.0815 4.861e-15 ***
---
Signif. codes:  0 '***' 0.001 '**' 0.01 '*' 0.05 '.' 0.1 ' ' 1

Residual standard error:

 0.01217955 (8 degrees of freedom)
```

```
plot(wmod)
```

```
AIC(wmod)
```

```
[1] -66.60298
```

#### GAM

```
Region<- "8"
```

```
gmod <- ndom_gam(r=Region,
               variant_int="XBB.1.5",
               startdate=startdate)

summary(gmod)
```

```
Family: gaussian 
Link function: identity 

Formula:
share ~ s(t)

Parametric coefficients:
            Estimate Std. Error t value Pr(>|t|)    
(Intercept) 0.283323   0.002547   111.2 2.01e-05 ***
---
Signif. codes:  0 '***' 0.001 '**' 0.01 '*' 0.05 '.' 0.1 ' ' 1

Approximate significance of smooth terms:
       edf Ref.df    F p-value    
s(t) 8.653  8.966 2030 0.00049 ***
---
Signif. codes:  0 '***' 0.001 '**' 0.01 '*' 0.05 '.' 0.1 ' ' 1

R-sq.(adj) =  0.999   Deviance explained =  100%
GCV = 0.00039801  Scale est. = 7.7857e-05  n = 12
```

```
plot(gmod)
```

```
AIC(gmod)
```

```
[1] -77.74693
```

```
rootfinder(mod = gmod,Range=c(1,150))
```

```
$root
[1] 107.949

$f.root
[1] 2.471299e-09

$iter
[1] 8

$init.it
[1] NA

$estim.prec
[1] 6.103516e-05
```

#### Compile

```
ttd(modl=lmod,
    modw=wmod,
    modg=gmod,
    Region=Region,
    variant = "XBB.1.5")->ttdx8
```

#### MSE

```
Variant<- "XBB.1.5"
methodsu<-data.frame(Variant=Variant,
                     Region=Region,
                     t=gmod$model$t,
                    share=gmod$model$share,
                    y=gmod$fitted.values,
                    logistic=c(lmod$predres[,1]),
                    weibull=c(wmod$predres[,1])
                    ) 

MSE_XBB<- bind_rows(MSE_XBB,methodsu)
```

### Region 9

#### logistic

```
lmod <- ndom_l(r="9",
               variant_int="XBB.1.5",
               startdate=startdate)

summary(lmod)
```

```
Model fitted: Generalised logistic (ED50 as parameter) (5 parms)

Parameter estimates:

                 Estimate  Std. Error t-value   p-value    
b:(Intercept) -0.05195559  0.00586791 -8.8542 2.089e-05 ***
c:(Intercept) -0.00058953  0.00515179 -0.1144  0.911714    
d:(Intercept)  0.76850903  0.01692833 45.3978 6.122e-11 ***
e:(Intercept) 81.76297825 17.26962671  4.7345  0.001474 ** 
f:(Intercept)  3.53603414  2.43252716  1.4536  0.184120    
---
Signif. codes:  0 '***' 0.001 '**' 0.01 '*' 0.05 '.' 0.1 ' ' 1

Residual standard error:

 0.01102255 (8 degrees of freedom)
```

```
plot(lmod)
```

```
AIC(lmod)
```

```
[1] -74.62232
```

#### Weibull

```
wmod <- ndom_w(r="9",
               variant_int="XBB.1.5",
               startdate=startdate)

summary(wmod)
```

```
Model fitted: Weibull (type 1) (4 parms)

Parameter estimates:

                 Estimate  Std. Error  t-value   p-value    
b:(Intercept) -4.2336e+00  3.4515e-01 -12.2658 6.388e-07 ***
c:(Intercept)  9.8533e-04  5.3485e-03   0.1842    0.8579    
d:(Intercept)  8.5953e-01  3.1977e-02  26.8796 6.608e-10 ***
e:(Intercept)  1.0505e+02  1.2210e+00  86.0427 1.957e-14 ***
---
Signif. codes:  0 '***' 0.001 '**' 0.01 '*' 0.05 '.' 0.1 ' ' 1

Residual standard error:

 0.01272092 (9 degrees of freedom)
```

```
plot(wmod)
```

```
AIC(wmod)
```

```
[1] -71.36521
```

#### GAM

```
Region<- "9"
```

```
gmod <- ndom_gam(r=Region,
               variant_int="XBB.1.5",
               startdate=startdate)

summary(gmod)
```

```
Family: gaussian 
Link function: identity 

Formula:
share ~ s(t)

Parametric coefficients:
            Estimate Std. Error t value Pr(>|t|)    
(Intercept) 0.258549   0.003019   85.64  5.8e-09 ***
---
Signif. codes:  0 '***' 0.001 '**' 0.01 '*' 0.05 '.' 0.1 ' ' 1

Approximate significance of smooth terms:
       edf Ref.df    F p-value    
s(t) 7.106  8.158 1146  <2e-16 ***
---
Signif. codes:  0 '***' 0.001 '**' 0.01 '*' 0.05 '.' 0.1 ' ' 1

R-sq.(adj) =  0.999   Deviance explained = 99.9%
GCV = 0.00031474  Scale est. = 0.00011849  n = 13
```

```
plot(gmod)
```

```
AIC(gmod)
```

```
[1] -75.12497
```

```
rootfinder(mod = gmod,Range=c(1,150))
```

```
$root
[1] 120.9898

$f.root
[1] 1.422829e-09

$iter
[1] 7

$init.it
[1] NA

$estim.prec
[1] 6.103516e-05
```

#### Compile

```
ttd(modl=lmod,
    modw=wmod,
    modg=gmod,
    Region=Region,
    variant = "XBB.1.5")->ttdx9
```

#### MSE

```
Variant<- "XBB.1.5"
methodsu<-data.frame(Variant=Variant,
                     Region=Region,
                     t=gmod$model$t,
                    share=gmod$model$share,
                    y=gmod$fitted.values,
                    logistic=c(lmod$predres[,1]),
                    weibull=c(wmod$predres[,1])
                    ) 

MSE_XBB<- bind_rows(MSE_XBB,methodsu)
```

### Region 10

#### logistic

```
lmod <- ndom_l(r="10",
               variant_int="XBB.1.5",
               startdate=startdate)

summary(lmod)
```

```
Model fitted: Generalised logistic (ED50 as parameter) (5 parms)

Parameter estimates:

                 Estimate  Std. Error  t-value   p-value    
b:(Intercept) -8.5606e-02  4.7018e-03 -18.2070 8.512e-08 ***
c:(Intercept) -7.7985e-04  2.1405e-03  -0.3643    0.7251    
d:(Intercept)  7.9969e-01  5.3931e-03 148.2799 4.836e-15 ***
e:(Intercept)  1.2021e+02  1.9973e+00  60.1854 6.454e-12 ***
f:(Intercept)  8.9040e-01  1.1092e-01   8.0277 4.260e-05 ***
---
Signif. codes:  0 '***' 0.001 '**' 0.01 '*' 0.05 '.' 0.1 ' ' 1

Residual standard error:

 0.004387375 (8 degrees of freedom)
```

```
plot(lmod)
```

```
AIC(lmod)
```

```
[1] -98.57383
```

#### Weibull

```
wmod <- ndom_w(r="10",
               variant_int="XBB.1.5",
               startdate=startdate)

summary(wmod)
```

```
Model fitted: Weibull (type 1) (4 parms)

Parameter estimates:

                 Estimate  Std. Error t-value   p-value    
b:(Intercept)  -5.3197867   0.6655870 -7.9926 2.230e-05 ***
c:(Intercept)   0.0032444   0.0080145  0.4048    0.6951    
d:(Intercept)   0.9088774   0.0472516 19.2349 1.280e-08 ***
e:(Intercept) 112.6509207   1.4875509 75.7291 6.175e-14 ***
---
Signif. codes:  0 '***' 0.001 '**' 0.01 '*' 0.05 '.' 0.1 ' ' 1

Residual standard error:

 0.02011652 (9 degrees of freedom)
```

```
plot(wmod)
```

```
AIC(wmod)
```

```
[1] -59.44958
```

#### GAM

```
Region<- "10"
```

```
gmod <- ndom_gam(r=Region,
               variant_int="XBB.1.5",
               startdate=startdate)

summary(gmod)
```

```
Family: gaussian 
Link function: identity 

Formula:
share ~ s(t)

Parametric coefficients:
             Estimate Std. Error t value Pr(>|t|)    
(Intercept) 0.2507517  0.0009338   268.5 1.77e-08 ***
---
Signif. codes:  0 '***' 0.001 '**' 0.01 '*' 0.05 '.' 0.1 ' ' 1

Approximate significance of smooth terms:
       edf Ref.df     F p-value    
s(t) 8.599  8.954 12216  <2e-16 ***
---
Signif. codes:  0 '***' 0.001 '**' 0.01 '*' 0.05 '.' 0.1 ' ' 1

R-sq.(adj) =      1   Deviance explained =  100%
GCV = 4.3324e-05  Scale est. = 1.1335e-05  n = 13
```

```
plot(gmod)
```

```
AIC(gmod)
```

```
[1] -107.38
```

```
rootfinder(mod = gmod,Range=c(1,150))
```

```
$root
[1] 124.7268

$f.root
[1] -4.922753e-10

$iter
[1] 7

$init.it
[1] NA

$estim.prec
[1] 6.103516e-05
```

#### Compile

```
ttd(modl=lmod,
    modw=wmod,
    modg=gmod,
    Region=Region,
    variant = "XBB.1.5")->ttdx10
```

#### MSE

```
Variant<- "XBB.1.5"
methodsu<-data.frame(Variant=Variant,
                     Region=Region,
                     t=gmod$model$t,
                    share=gmod$model$share,
                    y=gmod$fitted.values,
                    logistic=c(lmod$predres[,1]),
                    weibull=c(wmod$predres[,1])
                    ) 

MSE_XBB<- bind_rows(MSE_XBB,methodsu)
```

# Finding the maximum share of the previous variant.

```
covid |> filter(week=="2023-09-02") |> group_by(region) |> slice_max(share) |> 
  select(region,share,variant) |> 
  rename(prev_share=share,
         Region=region,
         prev_variant=variant) |> 
  mutate(Variant="JN.1") -> jn1_prev

covid |> filter(week=="2022-10-15") |> group_by(region) |> slice_max(share) |> 
  select(region,share,variant) |> 
  rename(prev_share=share,
         Region=region,
         prev_variant=variant) |> 
  mutate(Variant="XBB.1.5") -> xbb15_prev

prev <- bind_rows(jn1_prev,xbb15_prev)
```

# Importing Weekly Testing Data

```
read.csv("PctWeeklyPos_COVID.csv") |> 
  mutate(across(c("Region","Variant"),~as.factor(.x)))-> pct_weekly
```

# Combining all data

```
ttd <- bind_rows(ttdju,ttdj1,ttdj2,ttdj3,ttdj4,ttdj5,ttdj6,ttdj7,ttdj8,ttdj9,ttdj10,
                 ttdxu,ttdx1,ttdx2,ttdx3,ttdx4,ttdx5,ttdx6,ttdx7,ttdx8,ttdx9,ttdx10#,
#                 ttdeu,ttde1,ttde2,ttde3,ttde4,ttde5,ttde6,ttde7,ttde8,ttde9,ttde10,
#                 ttdhu,ttdh1,ttdh2,ttdh3,ttdh4,ttdh5,ttdh6,ttdh7,ttdh8,ttdh9,ttdh10
) |> 
  left_join(prev,by=c("Region","Variant")) |> 
  left_join(pct_weekly,by=c("Region","Variant")) |> 
    mutate(logVolume=log(TestVolume)) |> 
    mutate(Region = factor(Region,levels=c("USA","1","2","3","4",
                                                        "5","6","7","8","9","10")))

ttd |> 
  mutate(across(AIC_l:g25,~round(.x,2))) |>
  group_by(Region,Variant) |> 
  mutate(ttd_mean = mean(c(g50,l50,w50))) |> 
  ungroup() |> 
  DT::datatable()
```

```
MSE <- bind_rows(MSE_JN1[-1,],MSE_XBB[-1,]) |> rename(GAM=y) |> 
  mutate(SE_GAM = (GAM-share)^2,
         SE_Logistic = (logistic-share)^2,
         SE_Weibull = (weibull-share)^2) |> 
  group_by(Variant,Region) |> 
  summarize(MSE_GAM = mean(SE_GAM),
            MSE_Logistic = mean(SE_Logistic),
            MSE_Weibull = mean(SE_Weibull)) |> 
  ungroup()  |> 
    mutate(Region = factor(Region,levels=c("USA","1","2","3","4",
                                                        "5","6","7","8","9","10")))
```

```
`summarise()` has grouped output by 'Variant'. You can override using the
`.groups` argument.
```

```
MSE |> mutate(across(MSE_GAM:MSE_Weibull,~round(.x,5))) |> DT::datatable()
```

# Graphs

## AIC

```
ttd |> pivot_longer(cols=AIC_l:AIC_g,
                    names_prefix="AIC_",
                    names_to = "FitMethod",
                    values_to="AIC") |> 
  select(Region,Variant,FitMethod,AIC) ->aicplot


ggplot(aicplot,aes(x=Region,y=-AIC,group=FitMethod,fill=FitMethod)) + 
  geom_bar(position="dodge",stat="identity") + 
  scale_fill_brewer(palette="Set2",
                              name = "Fit Method",
                              label= c("GAM","Logistic", "Weibull")) + 
  theme_bw() + facet_wrap(~Variant,nrow=2)
```

```
ggsave("images/AICplot_bar.png")
```

```
Saving 7 x 5 in image
```

```
ggplot(aicplot |> filter(Variant %in% c("JN.1","XBB.1.5")),aes(x=Region,y=-AIC,group=FitMethod,fill=FitMethod)) + 
  geom_bar(position="dodge",stat="identity") + 
  scale_fill_brewer(palette="Set2",
                              name = "Fit Method",
                              label= c("GAM","Logistic", "Weibull")) + 
  theme_bw() + facet_wrap(~Variant,nrow=2)
```

```
ggsave("images/AICplot_bar_2variants.png")
```

```
Saving 7 x 5 in image
```

```
ggplot(aicplot,aes(x=Region,y=-AIC,group=FitMethod)) + 
  geom_point(aes(shape=FitMethod),size=3) + 
  scale_shape_discrete(name = "Fit Method",
                              label= c("GAM","Logistic", "Weibull")) + 
  theme_bw() + facet_wrap(~Variant,nrow=2) + geom_line()
```

```
ggsave("images/AICplot_scatter.png")
```

```
Saving 7 x 5 in image
```

```
ggplot(aicplot|> filter(Variant %in% c("JN.1","XBB.1.5")),aes(x=Region,y=-AIC,group=FitMethod)) + 
  geom_point(aes(shape=FitMethod),size=3) + 
  scale_shape_discrete(name = "Fit Method",
                              label= c("GAM","Logistic", "Weibull")) + 
  theme_bw() + facet_wrap(~Variant,nrow=2) + geom_line()
```

```
ggsave("images/AICplot_scatter_2variants.png")
```

```
Saving 7 x 5 in image
```

## MSE

```
MSE |> pivot_longer(cols=MSE_GAM:MSE_Weibull,
                    names_prefix="MSE_",
                    names_to = "FitMethod",
                    values_to="MSE") |> 
  select(Region,Variant,FitMethod,MSE) |> 
  mutate(RMSE=sqrt(MSE))->mseplot


ggplot(mseplot,aes(x=Region,y=RMSE,group=FitMethod,fill=FitMethod)) + 
  geom_bar(position="dodge",stat="identity") + 
  scale_fill_brewer(palette="Set2",
                              name = "Fit Method",
                              label= c("GAM","Logistic", "Weibull")) + 
  theme_bw() + facet_wrap(~Variant,nrow=2)
```

```
ggsave("images/MSEplot_bar.png")
```

```
Saving 7 x 5 in image
```

```
ggplot(mseplot |> filter(Variant %in% c("JN.1","XBB.1.5")),aes(x=Region,y=RMSE,group=FitMethod,fill=FitMethod)) + 
  geom_bar(position="dodge",stat="identity") + 
  scale_fill_brewer(palette="Set2",
                              name = "Fit Method",
                              label= c("GAM","Logistic", "Weibull")) + 
  theme_bw() + facet_wrap(~Variant,nrow=2)
```

```
ggsave("images/MSEplot_bar_2variants.png")
```

```
Saving 7 x 5 in image
```

```
ggplot(mseplot,aes(x=Region,y=RMSE,group=FitMethod)) + 
  geom_point(aes(shape=FitMethod),size=3) + 
  scale_shape_discrete(name = "Fit Method",
                              label= c("GAM","Logistic", "Weibull")) + 
  theme_bw() + facet_wrap(~Variant,nrow=2) + geom_line()
```

```
ggsave("images/MSEplot_scatter.png")
```

```
Saving 7 x 5 in image
```

```
ggplot(mseplot|> filter(Variant %in% c("JN.1","XBB.1.5")),aes(x=Region,y=RMSE,group=FitMethod)) + 
  geom_point(aes(shape=FitMethod),size=3) + 
  scale_shape_discrete(name = "Fit Method",
                              label= c("GAM","Logistic", "Weibull")) + 
  theme_bw() + facet_wrap(~Variant,nrow=2) + geom_line()
```

```
ggsave("images/MSEplot_scatter_2variants.png")
```

```
Saving 7 x 5 in image
```

## TTD

```
ttd |> gather(v,value,g50:g25) |> 
  separate(v,c("var","col")) |> 
  arrange(Region,Variant) |> 
  spread(col,value) |> 
  rename(TTD="<NA>") |> 
  # select(Region,Variant,var,lcl,ucl,TTD)-> ttdplot
  select(Region,Variant,var,TTD)-> ttdplot
```

```
Warning: Expected 2 pieces. Missing pieces filled with `NA` in 132 rows [1, 2, 3, 4, 5,
6, 7, 8, 9, 10, 11, 12, 13, 14, 15, 16, 17, 18, 19, 20, ...].
```

```
ttd50 <- ttdplot |> filter(grepl("50",var))

c(`l50`="Logistic", `w50`="Weibull",`g50`="GAM")->fitmethods

ggplot(ttd50,aes(x=Region,y=TTD,fill=Variant)) + 
  geom_bar(stat="identity",position="dodge") +
  #geom_errorbar(aes(ymin=lcl,ymax=ucl),position="dodge")+ ylim(c(0,300))+
    scale_fill_brewer(palette="Set2") + 
  theme_bw() + facet_wrap(~var,labeller=as_labeller(fitmethods),
                          nrow=3)
```

```
ggsave("images/TTDplot_bar.png")
```

```
Saving 7 x 5 in image
```

```
ggplot(ttd50 |> filter(Variant %in% c("JN.1","XBB.1.5")),aes(x=Region,y=TTD,fill=Variant)) + 
  geom_bar(stat="identity",position="dodge") +
#  geom_errorbar(aes(ymin=lcl,ymax=ucl),position="dodge")+ ylim(c(0,300))+
    scale_fill_brewer(palette="Set2") + 
  theme_bw() + facet_wrap(~var,labeller=as_labeller(fitmethods),
                          nrow=3)
```

```
ggsave("images/TTDplot_bar2variants.png")
```

```
Saving 7 x 5 in image
```

```
ggplot(ttd50 |> filter(Variant %in% c("JN.1","XBB.1.5")),aes(x=Region,y=TTD,fill=var)) + 
  geom_bar(stat="identity",position="dodge") +
#  geom_errorbar(aes(ymin=lcl,ymax=ucl),position="dodge")+ ylim(c(0,300))+
    scale_fill_brewer(palette="Set2",
                      label=fitmethods,
                      name="Method") + 
  theme_bw() + facet_wrap(~Variant,
                          nrow=3)
```

```
ggsave("images/TTDplot_bar_methods.png")
```

```
Saving 7 x 5 in image
```

```
ggplot(ttd50,aes(x=Region,y=TTD,fill=Variant,group=Variant)) +
  geom_point(aes(shape=Variant),size=2) + geom_line()+ 
#  geom_errorbar(aes(ymin=lcl,ymax=ucl),width=0.5)+ylim(c(0,300))+
  theme_bw() + facet_wrap(~var,labeller=as_labeller(fitmethods),
                          nrow=3)
```

```
ggsave("images/TTDplot_point.png")
```

```
Saving 7 x 5 in image
```

```
ggplot(ttd50 |> filter(Variant %in% c("JN.1","XBB.1.5")),aes(x=Region,y=TTD,fill=Variant)) + 
  geom_bar(stat="identity",position="dodge") +
# geom_errorbar(aes(ymin=lcl,ymax=ucl),position="dodge")+ ylim(c(0,300))+
    scale_fill_brewer(palette="Set2") + 
  theme_bw() + facet_wrap(~var,labeller=as_labeller(fitmethods),
                          nrow=3)
```

```
ggsave("images/TTDplot_bar2variants.png")
```

```
Saving 7 x 5 in image
```

```
ttd25 <- ttdplot |> filter(grepl("25",var))

c(`l25`="Logistic", `w25`="Weibull",`g25`="GAM")->fitmethods

ggplot(ttd25,aes(x=Region,y=TTD,fill=Variant)) + 
  geom_bar(stat="identity",position="dodge") +
  #geom_errorbar(aes(ymin=lcl,ymax=ucl),position="dodge")+ ylim(c(0,300))+
    scale_fill_brewer(palette="Set2") + 
  theme_bw() + facet_wrap(~var,labeller=as_labeller(fitmethods),
                          nrow=3)
```

```
ggsave("images/TTDplot25_bar.png")
```

```
Saving 7 x 5 in image
```

```
ggplot(ttd25,aes(x=Region,y=TTD,fill=Variant,group=Variant)) +
  geom_point(aes(shape=Variant),size=2) + geom_line()+ 
#  geom_errorbar(aes(ymin=lcl,ymax=ucl),width=0.5)+ylim(c(0,300))+
  theme_bw() + facet_wrap(~var,labeller=as_labeller(fitmethods),
                          nrow=3)
```

```
ggsave("images/TTDplot25_point.png")
```

```
Saving 7 x 5 in image
```

### Logistic only

```
ttd50 <- ttdplot |> filter(grepl("l50",var))


ggplot(ttd50,aes(x=Region,y=TTD,fill=Variant)) + 
  geom_bar(stat="identity",position="dodge") +
 # geom_errorbar(aes(ymin=lcl,ymax=ucl),position="dodge")+
  # ylim(c(0,300))+
    scale_fill_brewer(palette="Set2") + 
  theme_bw()
```

```
ggsave("images/logisticTTDplot_bar.png")
```

```
Saving 7 x 5 in image
```

```
ggplot(ttd50|> filter(Variant %in% c("JN.1","XBB.1.5")),aes(x=Region,y=TTD,fill=Variant)) + 
  geom_bar(stat="identity",position="dodge") +
#  geom_errorbar(aes(ymin=lcl,ymax=ucl),position="dodge")+
  # ylim(c(0,300))+
    scale_fill_brewer(palette="Set2") + 
  theme_bw()
```

```
ggsave("images/logisticTTDplot_bar_2variants.png")
```

```
Saving 7 x 5 in image
```

```
ggplot(ttd50,aes(x=Region,y=TTD,fill=Variant,group=Variant)) +
  geom_point(aes(shape=Variant),size=2) + geom_line()+ 
# geom_errorbar(aes(ymin=lcl,ymax=ucl),width=0.5)+
  # ylim(c(0,300))+
  theme_bw()
```

```
ggsave("images/logisticTTDplot_point.png")
```

```
Saving 7 x 5 in image
```

```
ggplot(ttd50|> filter(Variant %in% c("JN.1","XBB.1.5")),aes(x=Region,y=TTD,fill=Variant,group=Variant)) +
  geom_point(aes(shape=Variant),size=2) + geom_line()+ 
# geom_errorbar(aes(ymin=lcl,ymax=ucl),width=0.5)+
  # ylim(c(0,300))+
  theme_bw()
```

```
ggsave("images/logisticTTDplot_point2variants.png")
```

```
Saving 7 x 5 in image
```

```
ttd25 <- ttdplot |> filter(grepl("l25",var))


ggplot(ttd25,aes(x=Region,y=TTD,fill=Variant)) + 
  geom_bar(stat="identity",position="dodge") +
#  geom_errorbar(aes(ymin=lcl,ymax=ucl),position="dodge")+
  # ylim(c(0,300))+
    scale_fill_brewer(palette="Set2") + 
  theme_bw()
```

```
ggsave("images/logisticTTDplot25_bar.png")
```

```
Saving 7 x 5 in image
```

```
ggplot(ttd25,aes(x=Region,y=TTD,fill=Variant,group=Variant)) +
  geom_point(aes(shape=Variant),size=2) + geom_line()+ 
# geom_errorbar(aes(ymin=lcl,ymax=ucl),width=0.5)+
  # ylim(c(0,300))+
  theme_bw()
```

```
ggsave("images/logisticTTDplot25_point.png")
```

```
Saving 7 x 5 in image
```

## Combined Graph for USA

### JN.1

```
startdate <- as.Date("2023-09-02")
```

#### logistic

```
lmod <- ndom_l(r="USA",
               variant_int="JN.1",
               startdate=startdate)
```

#### Weibull

```
wmod <- ndom_w(r="USA",
               variant_int="JN.1",
               startdate=startdate)
```

#### GAM

```
Region<- "USA"
```

```
gmod <- ndom_gam(r=Region,
               variant_int="JN.1",
               startdate=startdate)
```

```
dif<- length(gmod$fitted.values)-length(lmod$predres[,1])

methods<-data.frame(t=gmod$model$t,
                    share=gmod$model$share,
                    y=gmod$fitted.values,
                    logistic=c(lmod$predres[,1],rep(NA,dif)),
                    weibull=c(wmod$predres[,1],rep(NA,dif)))

ggplot(data=methods,aes(x=t))+geom_point(aes(y=share)) + geom_line(aes(y=y))+
  geom_line(aes(y=logistic),color="red") + 
  geom_line(aes(y=weibull),color="green") +
  geom_hline(aes(yintercept=0.5),color="blue",linetype="dashed")+
  theme_bw()
```

```
ggsave("images/Fit_USA_JN1.png")
```

```
Saving 7 x 5 in image
```

### XBB.1.5

```
startdate <- as.Date("2022-10-15")
```

#### logistic

```
lmod <- ndom_l(r="USA",
               variant_int="XBB.1.5",
               startdate=startdate)
```

#### Weibull

```
wmod <- ndom_w(r="USA",
               variant_int="XBB.1.5",
               startdate=startdate)
```

#### GAM

```
Region<- "USA"
```

```
gmod <- ndom_gam(r=Region,
               variant_int="XBB.1.5",
               startdate=startdate)
```

```
dif<- length(gmod$fitted.values)-length(lmod$predres[,1])

methods<-data.frame(t=gmod$model$t,
                    share=gmod$model$share,
                    y=gmod$fitted.values,
                    logistic=c(lmod$predres[,1],rep(NA,dif)),
                    weibull=c(wmod$predres[,1],rep(NA,dif)))

ggplot(data=methods,aes(x=t))+geom_point(aes(y=share)) + geom_line(aes(y=y))+
  geom_line(aes(y=logistic),color="red") + 
  geom_line(aes(y=weibull),color="green") +
  geom_hline(aes(yintercept=0.5),color="blue",linetype="dashed")+
  theme_bw() + 
  xlab("Time (Days)") + 
  ylab("Share")
```

```
ggsave("images/Fit_USA_XBB.png")
```

```
Saving 7 x 5 in image
```

### BA.5

```
startdate <- as.Date("2021-10-30")+140
```

#### logistic

```
lmod <- ndom_l(r="USA",
               variant_int="BA.5",
               startdate=startdate)
```

#### Weibull

```
wmod <- ndom_w(r="USA",
               variant_int="BA.5",
               startdate=startdate)
```

#### GAM

```
Region<- "USA"
```

```
gmod <- ndom_gam(r=Region,
               variant_int="BA.5",
               startdate=startdate)
```

```
dif<- length(gmod$fitted.values)-length(lmod$predres[,1])

methods<-data.frame(t=gmod$model$t,
                    share=gmod$model$share,
                    y=gmod$fitted.values,
                    logistic=c(lmod$predres[,1],rep(NA,dif)),
                    weibull=c(wmod$predres[,1],rep(NA,dif)))

ggplot(data=methods,aes(x=t))+geom_point(aes(y=share)) + geom_line(aes(y=y))+
  geom_line(aes(y=logistic),color="red") + 
  geom_line(aes(y=weibull),color="green") +
  geom_hline(aes(yintercept=0.5),color="blue",linetype="dashed")+
  theme_bw() + 
  xlab("Time (Days)") + 
  ylab("Share")
```

```
ggsave("images/Fit_USA_BA.png")
```

```
Saving 7 x 5 in image
```

```
data.frame(AIC_lmod=AIC(lmod),
           AIC_wmod=AIC(wmod),
           AIC_gmod=AIC(gmod),
           variant="BA.5",
           Region="USA")
```

```
   AIC_lmod  AIC_wmod  AIC_gmod variant Region
1 -115.2468 -57.26046 -103.8747    BA.5    USA
```

### EG.5

```
startdate <- as.Date("2023-03-18")
```

#### logistic

```
lmod <- ndom_l(r="USA",
               variant_int="EG.5",
               startdate=startdate)
```

#### Weibull

```
wmod <- ndom_w(r="USA",
               variant_int="EG.5",
               startdate=startdate)
```

#### GAM

```
Region<- "USA"
```

```
gmod <- ndom_gam(r=Region,
               variant_int="EG.5",
               startdate=startdate)
```

```
dif<- length(gmod$fitted.values)-length(lmod$predres[,1])

methods<-data.frame(t=gmod$model$t,
                    share=gmod$model$share,
                    y=gmod$fitted.values,
                    logistic=c(lmod$predres[,1],rep(NA,dif)),
                    weibull=c(wmod$predres[,1],rep(NA,dif)))

ggplot(data=methods,aes(x=t))+geom_point(aes(y=share)) + geom_line(aes(y=y))+
  geom_line(aes(y=logistic),color="red") + 
  geom_line(aes(y=weibull),color="green") +
  geom_hline(aes(yintercept=0.5),color="blue",linetype="dashed")+
  theme_bw() + 
  xlab("Time (Days)") + 
  ylab("Share")
```

```
ggsave("images/Fit_USA_EG.png")
```

```
Saving 7 x 5 in image
```

```
data.frame(AIC_lmod=AIC(lmod),
           AIC_wmod=AIC(wmod),
           AIC_gmod=AIC(gmod),
           variant="BA.5",
           Region="USA")
```

```
   AIC_lmod  AIC_wmod  AIC_gmod variant Region
1 -113.5918 -97.60637 -117.1909    BA.5    USA
```

## Combined Graph for Region 1

```
Region<- "1"
```

### JN.1

```
startdate <- as.Date("2023-09-02")
```

#### logistic

```
lmod <- ndom_l(r=Region,
               variant_int="JN.1",
               startdate=startdate)
```

#### Weibull

```
wmod <- ndom_w(r=Region,
               variant_int="JN.1",
               startdate=startdate)
```

#### GAM

```
gmod <- ndom_gam(r=Region,
               variant_int="JN.1",
               startdate=startdate)
```

```
dif<- length(gmod$fitted.values)-length(lmod$predres[,1])

methods<-data.frame(t=gmod$model$t,
                    share=gmod$model$share,
                    y=gmod$fitted.values,
                    logistic=c(lmod$predres[,1],rep(NA,dif)),
                    weibull=c(wmod$predres[,1],rep(NA,dif)))

ggplot(data=methods,aes(x=t))+geom_point(aes(y=share)) + geom_line(aes(y=y))+
  geom_line(aes(y=logistic),color="red") + 
  geom_line(aes(y=weibull),color="green") +
  geom_hline(aes(yintercept=0.5),color="blue",linetype="dashed")+
  theme_bw() + 
  xlab("Time (Days)") + 
  ylab("Share")
```

```
ggsave("images/Fit_1_JN1.png")
```

```
Saving 7 x 5 in image
```

### XBB.1.5

```
startdate <- as.Date("2022-10-15")
```

#### logistic

```
lmod <- ndom_l(r=Region,
               variant_int="XBB.1.5",
               startdate=startdate)
```

#### Weibull

```
wmod <- ndom_w(r=Region,
               variant_int="XBB.1.5",
               startdate=startdate)
```

#### GAM

```
gmod <- ndom_gam(r=Region,
               variant_int="XBB.1.5",
               startdate=startdate)
```

```
dif<- length(gmod$fitted.values)-length(lmod$predres[,1])

methods<-data.frame(t=gmod$model$t,
                    share=gmod$model$share,
                    y=gmod$fitted.values,
                    logistic=c(lmod$predres[,1],rep(NA,dif)),
                    weibull=c(wmod$predres[,1],rep(NA,dif)))

ggplot(data=methods,aes(x=t))+geom_point(aes(y=share)) + geom_line(aes(y=y))+
  geom_line(aes(y=logistic),color="red") + 
  geom_line(aes(y=weibull),color="green") +
  geom_hline(aes(yintercept=0.5),color="blue",linetype="dashed")+
  theme_bw() + 
  xlab("Time (Days)") + 
  ylab("Share")
```

```
ggsave("images/Fit_1_XBB.png")
```

```
Saving 7 x 5 in image
```

## Combined Graph for Region 2

```
Region<- "2"
```

### JN.1

```
startdate <- as.Date("2023-09-02")
```

#### logistic

```
lmod <- ndom_l(r=Region,
               variant_int="JN.1",
               startdate=startdate)
```

#### Weibull

```
wmod <- ndom_w(r=Region,
               variant_int="JN.1",
               startdate=startdate)
```

#### GAM

```
gmod <- ndom_gam(r=Region,
               variant_int="JN.1",
               startdate=startdate)
```

```
dif<- length(gmod$fitted.values)-length(lmod$predres[,1])

methods<-data.frame(t=gmod$model$t,
                    share=gmod$model$share,
                    y=gmod$fitted.values,
                    logistic=c(lmod$predres[,1],rep(NA,dif)),
                    weibull=c(wmod$predres[,1],rep(NA,dif)))

ggplot(data=methods,aes(x=t))+geom_point(aes(y=share)) + geom_line(aes(y=y))+
  geom_line(aes(y=logistic),color="red") + 
  geom_line(aes(y=weibull),color="green") +
  geom_hline(aes(yintercept=0.5),color="blue",linetype="dashed")+
  theme_bw() + 
  xlab("Time (Days)") + 
  ylab("Share")
```

```
ggsave("images/Fit_2_JN1.png")
```

```
Saving 7 x 5 in image
```

### XBB.1.5

```
startdate <- as.Date("2022-10-15")
```

#### logistic

```
lmod <- ndom_l(r=Region,
               variant_int="XBB.1.5",
               startdate=startdate)
```

#### Weibull

```
wmod <- ndom_w(r=Region,
               variant_int="XBB.1.5",
               startdate=startdate)
```

#### GAM

```
gmod <- ndom_gam(r=Region,
               variant_int="XBB.1.5",
               startdate=startdate)
```

```
dif<- length(gmod$fitted.values)-length(lmod$predres[,1])

methods<-data.frame(t=gmod$model$t,
                    share=gmod$model$share,
                    y=gmod$fitted.values,
                    logistic=c(lmod$predres[,1],rep(NA,dif)),
                    weibull=c(wmod$predres[,1],rep(NA,dif)))

ggplot(data=methods,aes(x=t))+geom_point(aes(y=share)) + geom_line(aes(y=y))+
  geom_line(aes(y=logistic),color="red") + 
  geom_line(aes(y=weibull),color="green") +
  geom_hline(aes(yintercept=0.5),color="blue",linetype="dashed")+
  theme_bw() + 
  xlab("Time (Days)") + 
  ylab("Share")
```

```
ggsave("images/Fit_2_XBB.png")
```

```
Saving 7 x 5 in image
```

## Combined Graph for Region 5

```
Region<- "5"
```

### JN.1

```
startdate <- as.Date("2023-09-02")
```

#### logistic

```
lmod <- ndom_l(r=Region,
               variant_int="JN.1",
               startdate=startdate)
```

#### Weibull

```
wmod <- ndom_w(r=Region,
               variant_int="JN.1",
               startdate=startdate)
```

#### GAM

```
gmod <- ndom_gam(r=Region,
               variant_int="JN.1",
               startdate=startdate)
```

```
dif<- length(gmod$fitted.values)-length(lmod$predres[,1])

methods<-data.frame(t=gmod$model$t,
                    share=gmod$model$share,
                    y=gmod$fitted.values,
                    logistic=c(lmod$predres[,1],rep(NA,dif)),
                    weibull=c(wmod$predres[,1],rep(NA,dif)))

ggplot(data=methods,aes(x=t))+geom_point(aes(y=share)) + geom_line(aes(y=y))+
  geom_line(aes(y=logistic),color="red") + 
  geom_line(aes(y=weibull),color="green") +
  geom_hline(aes(yintercept=0.5),color="blue",linetype="dashed")+
  theme_bw() + 
  xlab("Time (Days)") + 
  ylab("Share")
```

```
ggsave("images/Fit_5_JN1.png")
```

```
Saving 7 x 5 in image
```

### XBB.1.5

```
startdate <- as.Date("2022-10-15")
```

#### logistic

```
lmod <- ndom_l(r=Region,
               variant_int="XBB.1.5",
               startdate=startdate)
```

#### Weibull

```
wmod <- ndom_w(r=Region,
               variant_int="XBB.1.5",
               startdate=startdate)
```

#### GAM

```
gmod <- ndom_gam(r=Region,
               variant_int="XBB.1.5",
               startdate=startdate)
```

```
dif<- length(gmod$fitted.values)-length(lmod$predres[,1])

methods<-data.frame(t=gmod$model$t,
                    share=gmod$model$share,
                    y=gmod$fitted.values,
                    logistic=c(lmod$predres[,1],rep(NA,dif)),
                    weibull=c(wmod$predres[,1],rep(NA,dif)))

ggplot(data=methods,aes(x=t))+geom_point(aes(y=share)) + geom_line(aes(y=y))+
  geom_line(aes(y=logistic),color="red") + 
  geom_line(aes(y=weibull),color="green") +
  geom_hline(aes(yintercept=0.5),color="blue",linetype="dashed")+
  theme_bw() + 
  xlab("Time (Days)") + 
  ylab("Share")
```

```
ggsave("images/Fit_5_XBB.png")
```

```
Saving 7 x 5 in image
```

# Combined Analysis of Logistic Estimates [ED 50]

## USA

```
r="USA"
```

```
startdate <- as.Date("2023-09-02")

variant_int <- "JN.1"

var_data_jn1 <- covid |> 
  filter(usa_or_hhsregion==r) |> 
  filter(variant %in% c(variant_int)) |> 
  mutate(t = as.numeric(week-startdate)) |> 
  arrange(t)

var_data_jn1 |> dplyr::select(t,share) |>slice_max(share,n=1) -> maxshare

maxtime <- maxshare[1] |> as.numeric()

var_data_jn1 <- var_data_jn1 |> 
  filter(between(t,0,maxtime))

startdate <- as.Date("2022-10-15")

variant_int <- "XBB.1.5"

var_data_xbb <- covid |> 
  filter(usa_or_hhsregion==r) |> 
  filter(variant %in% c(variant_int)) |> 
  mutate(t = as.numeric(week-startdate)) |> 
  arrange(t)

var_data_xbb |> dplyr::select(t,share) |>slice_max(share,n=1) -> maxshare

maxtime <- maxshare[1] |> as.numeric()

var_data_xbb <- var_data_xbb |> 
  filter(between(t,0,maxtime))

startdate <- as.Date("2023-06-10")

variant_int <- "HV.1"

var_data_ba <- covid |> 
  filter(usa_or_hhsregion==r) |> 
  filter(variant %in% c(variant_int)) |> 
  mutate(t = as.numeric(week-startdate)) |> 
  arrange(t)

var_data_ba |> dplyr::select(t,share) |>slice_max(share,n=1) -> maxshare

maxtime <- maxshare[1] |> as.numeric()

var_data_ba <- var_data_ba |> 
  filter(between(t,0,maxtime))

startdate <- as.Date("2023-03-18")

variant_int <- "EG.5"

var_data_eg <- covid |> 
  filter(usa_or_hhsregion==r) |> 
  filter(variant %in% c(variant_int)) |> 
  mutate(t = as.numeric(week-startdate)) |> 
  arrange(t)

var_data_eg |> dplyr::select(t,share) |>slice_max(share,n=1) -> maxshare

maxtime <- maxshare[1] |> as.numeric()

var_data_eg <- var_data_eg |> 
  filter(between(t,0,maxtime))


var_data <- bind_rows(var_data_jn1,var_data_xbb,var_data_ba,var_data_eg)

lmod <- drc::drm(share~t,as.factor(variant),fct=drc::L.5(),data=var_data)
```

```
drc::EDcomp(lmod,c(10,10),interval="delta")
```

```
Estimated ratios of effect doses

                   Estimate   Lower   Upper
EG.5/HV.1:10/10     1.01449 0.89488 1.13411
EG.5/JN.1:10/10     0.95741 0.86815 1.04668
EG.5/XBB.1.5:10/10  1.13792 1.03042 1.24541
HV.1/JN.1:10/10     0.94373 0.87288 1.01458
HV.1/XBB.1.5:10/10  1.12166 1.03574 1.20758
JN.1/XBB.1.5:10/10  1.18854 1.15803 1.21904
```

```
drc::EDcomp(lmod,c(50,50),interval="delta")
```

```
Estimated ratios of effect doses

                   Estimate   Lower   Upper
EG.5/HV.1:50/50     0.98593 0.92023 1.05163
EG.5/JN.1:50/50     1.14490 1.11811 1.17169
EG.5/XBB.1.5:50/50  1.24255 1.21332 1.27177
HV.1/JN.1:50/50     1.16124 1.08841 1.23408
HV.1/XBB.1.5:50/50  1.26028 1.18118 1.33938
JN.1/XBB.1.5:50/50  1.08529 1.07785 1.09272
```

### GAM

```
gmod <- mgcv::gam(share~variant+s(t),data=var_data |> filter(variant%in%c("JN.1","XBB.1.5")))
summary(gmod)
```

```
Family: gaussian 
Link function: identity 

Formula:
share ~ variant + s(t)

Parametric coefficients:
               Estimate Std. Error t value Pr(>|t|)    
(Intercept)    0.244225   0.008939  27.322 4.75e-15 ***
variantXBB.1.5 0.045017   0.012641   3.561  0.00254 ** 
---
Signif. codes:  0 '***' 0.001 '**' 0.01 '*' 0.05 '.' 0.1 ' ' 1

Approximate significance of smooth terms:
       edf Ref.df   F p-value    
s(t) 5.693  6.848 354  <2e-16 ***
---
Signif. codes:  0 '***' 0.001 '**' 0.01 '*' 0.05 '.' 0.1 ' ' 1

R-sq.(adj) =  0.991   Deviance explained = 99.3%
GCV = 0.0014111  Scale est. = 0.0009588  n = 24
```

## Region 1

```
r="1"
```

```
startdate <- as.Date("2023-09-02")

variant_int <- "JN.1"

var_data_jn1 <- covid |> 
  filter(usa_or_hhsregion==r) |> 
  filter(variant %in% c(variant_int)) |> 
  mutate(t = as.numeric(week-startdate)) |> 
  arrange(t)

var_data_jn1 |> dplyr::select(t,share) |>slice_max(share,n=1) -> maxshare

maxtime <- maxshare[1] |> as.numeric()

var_data_jn1 <- var_data_jn1 |> 
  filter(between(t,0,maxtime))

startdate <- as.Date("2022-10-15")

variant_int <- "XBB.1.5"

var_data_xbb <- covid |> 
  filter(usa_or_hhsregion==r) |> 
  filter(variant %in% c(variant_int)) |> 
  mutate(t = as.numeric(week-startdate)) |> 
  arrange(t)

var_data_xbb |> dplyr::select(t,share) |>slice_max(share,n=1) -> maxshare

maxtime <- maxshare[1] |> as.numeric()

var_data_xbb <- var_data_xbb |> 
  filter(between(t,0,maxtime))

startdate <- as.Date("2023-06-10")

variant_int <- "HV.1"


var_data_ba <- covid |> 
  filter(usa_or_hhsregion==r) |> 
  filter(variant %in% c(variant_int)) |> 
  mutate(t = as.numeric(week-startdate)) |> 
  arrange(t)

var_data_ba |> dplyr::select(t,share) |>slice_max(share,n=1) -> maxshare

maxtime <- maxshare[1] |> as.numeric()

var_data_ba <- var_data_ba |> 
  filter(between(t,0,maxtime))

startdate <- as.Date("2023-03-18")

variant_int <- "EG.5"

var_data_eg <- covid |> 
  filter(usa_or_hhsregion==r) |> 
  filter(variant %in% c(variant_int)) |> 
  mutate(t = as.numeric(week-startdate)) |> 
  arrange(t)

var_data_eg |> dplyr::select(t,share) |>slice_max(share,n=1) -> maxshare

maxtime <- maxshare[1] |> as.numeric()

var_data_eg <- var_data_eg |> 
  filter(between(t,0,maxtime))


var_data <- bind_rows(var_data_jn1,var_data_xbb,var_data_ba,var_data_eg)

lmod <- drc::drm(share~t,as.factor(variant),fct=drc::L.5(),data=var_data)
```

```
drc::EDcomp(lmod,c(10,10),interval="delta")
```

```
Estimated ratios of effect doses

                   Estimate    Lower    Upper
EG.5/HV.1:10/10     1.44650 -0.20385  3.09685
EG.5/JN.1:10/10     1.52552 -0.18539  3.23643
EG.5/XBB.1.5:10/10  2.44936 -0.36305  5.26177
HV.1/JN.1:10/10     1.05463  0.78301  1.32624
HV.1/XBB.1.5:10/10  1.69330  1.09004  2.29656
JN.1/XBB.1.5:10/10  1.60559  1.14292  2.06826
```

```
drc::EDcomp(lmod,c(50,50),interval="delta")
```

```
Estimated ratios of effect doses

                   Estimate   Lower   Upper
EG.5/HV.1:50/50     1.49852 0.36187 2.63516
EG.5/JN.1:50/50     1.64618 0.39924 2.89312
EG.5/XBB.1.5:50/50  2.10397 0.50796 3.69998
HV.1/JN.1:50/50     1.09854 1.04504 1.15204
HV.1/XBB.1.5:50/50  1.40403 1.31490 1.49317
JN.1/XBB.1.5:50/50  1.27809 1.21472 1.34147
```

### GAM

```
gmod <- mgcv::gam(share~variant+s(t),data=var_data |> filter(variant%in%c("JN.1","XBB.1.5")))
summary(gmod)
```

```
Family: gaussian 
Link function: identity 

Formula:
share ~ variant + s(t)

Parametric coefficients:
               Estimate Std. Error t value Pr(>|t|)    
(Intercept)     0.21433    0.02677   8.007 4.07e-07 ***
variantXBB.1.5  0.16945    0.04038   4.197 0.000627 ***
---
Signif. codes:  0 '***' 0.001 '**' 0.01 '*' 0.05 '.' 0.1 ' ' 1

Approximate significance of smooth terms:
       edf Ref.df     F p-value    
s(t) 3.291  4.094 67.24  <2e-16 ***
---
Signif. codes:  0 '***' 0.001 '**' 0.01 '*' 0.05 '.' 0.1 ' ' 1

R-sq.(adj) =  0.929   Deviance explained = 94.4%
GCV = 0.010998  Scale est. = 0.0083527  n = 22
```

## Region 2

```
r="2"
```

```
startdate <- as.Date("2023-09-02")

variant_int <- "JN.1"

var_data_jn1 <- covid |> 
  filter(usa_or_hhsregion==r) |> 
  filter(variant %in% c(variant_int)) |> 
  mutate(t = as.numeric(week-startdate)) |> 
  arrange(t)

var_data_jn1 |> dplyr::select(t,share) |>slice_max(share,n=1) -> maxshare

maxtime <- maxshare[1] |> as.numeric()

var_data_jn1 <- var_data_jn1 |> 
  filter(between(t,0,maxtime))

startdate <- as.Date("2022-10-15")

variant_int <- "XBB.1.5"

var_data_xbb <- covid |> 
  filter(usa_or_hhsregion==r) |> 
  filter(variant %in% c(variant_int)) |> 
  mutate(t = as.numeric(week-startdate)) |> 
  arrange(t)

var_data_xbb |> dplyr::select(t,share) |>slice_max(share,n=1) -> maxshare

maxtime <- maxshare[1] |> as.numeric()

var_data_xbb <- var_data_xbb |> 
  filter(between(t,0,maxtime))

startdate <- as.Date("2023-06-10")

variant_int <- "HV.1"


var_data_ba <- covid |> 
  filter(usa_or_hhsregion==r) |> 
  filter(variant %in% c(variant_int)) |> 
  mutate(t = as.numeric(week-startdate)) |> 
  arrange(t)

var_data_ba |> dplyr::select(t,share) |>slice_max(share,n=1) -> maxshare

maxtime <- maxshare[1] |> as.numeric()

var_data_ba <- var_data_ba |> 
  filter(between(t,0,maxtime))

startdate <- as.Date("2023-03-18")

variant_int <- "EG.5"

var_data_eg <- covid |> 
  filter(usa_or_hhsregion==r) |> 
  filter(variant %in% c(variant_int)) |> 
  mutate(t = as.numeric(week-startdate)) |> 
  arrange(t)

var_data_eg |> dplyr::select(t,share) |>slice_max(share,n=1) -> maxshare

maxtime <- maxshare[1] |> as.numeric()

var_data_eg <- var_data_eg |> 
  filter(between(t,0,maxtime))


var_data <- bind_rows(var_data_jn1,var_data_xbb,var_data_ba,var_data_eg)

lmod <- drc::drm(share~t,as.factor(variant),fct=drc::L.5(),data=var_data)
```

```
drc::EDcomp(lmod,c(10,10),interval="delta")
```

```
Estimated ratios of effect doses

                   Estimate   Lower   Upper
EG.5/HV.1:10/10     1.42070 1.18762 1.65379
EG.5/JN.1:10/10     1.43413 1.21366 1.65459
EG.5/XBB.1.5:10/10  2.21646 1.87361 2.55931
HV.1/JN.1:10/10     1.00945 0.93819 1.08071
HV.1/XBB.1.5:10/10  1.56011 1.44677 1.67345
JN.1/XBB.1.5:10/10  1.54551 1.47650 1.61452
```

```
drc::EDcomp(lmod,c(50,50),interval="delta")
```

```
Estimated ratios of effect doses

                   Estimate  Lower  Upper
EG.5/HV.1:50/50      1.5689 1.3316 1.8062
EG.5/JN.1:50/50      1.9771 1.7110 2.2431
EG.5/XBB.1.5:50/50   2.6573 2.2989 3.0157
HV.1/JN.1:50/50      1.2602 1.1725 1.3479
HV.1/XBB.1.5:50/50   1.6938 1.5749 1.8127
JN.1/XBB.1.5:50/50   1.3441 1.3273 1.3609
```

### GAM

```
gmod <- mgcv::gam(share~variant+s(t),data=var_data |> filter(variant%in%c("JN.1","XBB.1.5")))
summary(gmod)
```

```
Family: gaussian 
Link function: identity 

Formula:
share ~ variant + s(t)

Parametric coefficients:
               Estimate Std. Error t value Pr(>|t|)    
(Intercept)     0.26291    0.02666   9.860 1.38e-08 ***
variantXBB.1.5  0.16818    0.03877   4.338 0.000416 ***
---
Signif. codes:  0 '***' 0.001 '**' 0.01 '*' 0.05 '.' 0.1 ' ' 1

Approximate significance of smooth terms:
       edf Ref.df     F p-value    
s(t) 3.408  4.236 73.35  <2e-16 ***
---
Signif. codes:  0 '***' 0.001 '**' 0.01 '*' 0.05 '.' 0.1 ' ' 1

R-sq.(adj) =  0.935   Deviance explained = 94.8%
GCV = 0.011039  Scale est. = 0.0084435  n = 23
```

## Region 3

```
r="3"
```

```
startdate <- as.Date("2023-09-02")

variant_int <- "JN.1"

var_data_jn1 <- covid |> 
  filter(usa_or_hhsregion==r) |> 
  filter(variant %in% c(variant_int)) |> 
  mutate(t = as.numeric(week-startdate)) |> 
  arrange(t)

var_data_jn1 |> dplyr::select(t,share) |>slice_max(share,n=1) -> maxshare

maxtime <- maxshare[1] |> as.numeric()

var_data_jn1 <- var_data_jn1 |> 
  filter(between(t,0,maxtime))

startdate <- as.Date("2022-10-15")

variant_int <- "XBB.1.5"

var_data_xbb <- covid |> 
  filter(usa_or_hhsregion==r) |> 
  filter(variant %in% c(variant_int)) |> 
  mutate(t = as.numeric(week-startdate)) |> 
  arrange(t)

var_data_xbb |> dplyr::select(t,share) |>slice_max(share,n=1) -> maxshare

maxtime <- maxshare[1] |> as.numeric()

var_data_xbb <- var_data_xbb |> 
  filter(between(t,0,maxtime))

startdate <- as.Date("2023-06-10")

variant_int <- "HV.1"


var_data_ba <- covid |> 
  filter(usa_or_hhsregion==r) |> 
  filter(variant %in% c(variant_int)) |> 
  mutate(t = as.numeric(week-startdate)) |> 
  arrange(t)

var_data_ba |> dplyr::select(t,share) |>slice_max(share,n=1) -> maxshare

maxtime <- maxshare[1] |> as.numeric()

var_data_ba <- var_data_ba |> 
  filter(between(t,0,maxtime))

startdate <- as.Date("2023-03-18")

variant_int <- "EG.5"

var_data_eg <- covid |> 
  filter(usa_or_hhsregion==r) |> 
  filter(variant %in% c(variant_int)) |> 
  mutate(t = as.numeric(week-startdate)) |> 
  arrange(t)

var_data_eg |> dplyr::select(t,share) |>slice_max(share,n=1) -> maxshare

maxtime <- maxshare[1] |> as.numeric()

var_data_eg <- var_data_eg |> 
  filter(between(t,0,maxtime))


var_data <- bind_rows(var_data_jn1,var_data_xbb,var_data_ba,var_data_eg)

lmod <- drc::drm(share~t,as.factor(variant),fct=drc::L.5(),data=var_data)
```

```
drc::EDcomp(lmod,c(10,10),interval="delta")
```

```
Estimated ratios of effect doses

                   Estimate   Lower   Upper
EG.5/HV.1:10/10     1.10562 0.62303 1.58821
EG.5/JN.1:10/10     0.96836 0.76965 1.16708
EG.5/XBB.1.5:10/10  1.27594 1.01069 1.54119
HV.1/JN.1:10/10     0.87585 0.53251 1.21920
HV.1/XBB.1.5:10/10  1.15404 0.70001 1.60808
JN.1/XBB.1.5:10/10  1.31762 1.21245 1.42280
```

```
drc::EDcomp(lmod,c(50,50),interval="delta")
```

```
Estimated ratios of effect doses

                   Estimate   Lower   Upper
EG.5/HV.1:50/50     1.26890 0.80015 1.73765
EG.5/JN.1:50/50     1.40479 0.94307 1.86652
EG.5/XBB.1.5:50/50  1.71426 1.15094 2.27757
HV.1/JN.1:50/50     1.10710 0.91880 1.29539
HV.1/XBB.1.5:50/50  1.35098 1.12140 1.58056
JN.1/XBB.1.5:50/50  1.22029 1.19467 1.24591
```

### GAM

```
gmod <- mgcv::gam(share~variant+s(t),data=var_data |> filter(variant%in%c("JN.1","XBB.1.5")))
summary(gmod)
```

```
Family: gaussian 
Link function: identity 

Formula:
share ~ variant + s(t)

Parametric coefficients:
               Estimate Std. Error t value Pr(>|t|)    
(Intercept)     0.23753    0.02160  10.999 2.12e-09 ***
variantXBB.1.5  0.11708    0.03054   3.833  0.00123 ** 
---
Signif. codes:  0 '***' 0.001 '**' 0.01 '*' 0.05 '.' 0.1 ' ' 1

Approximate significance of smooth terms:
       edf Ref.df     F p-value    
s(t) 4.074  5.022 91.19  <2e-16 ***
---
Signif. codes:  0 '***' 0.001 '**' 0.01 '*' 0.05 '.' 0.1 ' ' 1

R-sq.(adj) =  0.953   Deviance explained = 96.4%
GCV = 0.0074934  Scale est. = 0.005597  n = 24
```

## Region 4

```
r="4"
```

```
startdate <- as.Date("2023-09-02")

variant_int <- "JN.1"

var_data_jn1 <- covid |> 
  filter(usa_or_hhsregion==r) |> 
  filter(variant %in% c(variant_int)) |> 
  mutate(t = as.numeric(week-startdate)) |> 
  arrange(t)

var_data_jn1 |> dplyr::select(t,share) |>slice_max(share,n=1) -> maxshare

maxtime <- maxshare[1] |> as.numeric()

var_data_jn1 <- var_data_jn1 |> 
  filter(between(t,0,maxtime))

startdate <- as.Date("2022-10-15")

variant_int <- "XBB.1.5"

var_data_xbb <- covid |> 
  filter(usa_or_hhsregion==r) |> 
  filter(variant %in% c(variant_int)) |> 
  mutate(t = as.numeric(week-startdate)) |> 
  arrange(t)

var_data_xbb |> dplyr::select(t,share) |>slice_max(share,n=1) -> maxshare

maxtime <- maxshare[1] |> as.numeric()

var_data_xbb <- var_data_xbb |> 
  filter(between(t,0,maxtime))

startdate <- as.Date("2023-06-10")

variant_int <- "HV.1"


var_data_ba <- covid |> 
  filter(usa_or_hhsregion==r) |> 
  filter(variant %in% c(variant_int)) |> 
  mutate(t = as.numeric(week-startdate)) |> 
  arrange(t)

var_data_ba |> dplyr::select(t,share) |>slice_max(share,n=1) -> maxshare

maxtime <- maxshare[1] |> as.numeric()

var_data_ba <- var_data_ba |> 
  filter(between(t,0,maxtime))

startdate <- as.Date("2023-03-18")

variant_int <- "EG.5"

var_data_eg <- covid |> 
  filter(usa_or_hhsregion==r) |> 
  filter(variant %in% c(variant_int)) |> 
  mutate(t = as.numeric(week-startdate)) |> 
  arrange(t)

var_data_eg |> dplyr::select(t,share) |>slice_max(share,n=1) -> maxshare

maxtime <- maxshare[1] |> as.numeric()

var_data_eg <- var_data_eg |> 
  filter(between(t,0,maxtime))


var_data <- bind_rows(var_data_jn1,var_data_xbb,var_data_ba,var_data_eg)

lmod <- drc::drm(share~t,as.factor(variant),fct=drc::L.5(),data=var_data)
```

```
drc::EDcomp(lmod,c(10,10),interval="delta")
```

```
Estimated ratios of effect doses

                   Estimate    Lower    Upper
EG.5/HV.1:10/10     0.92319 -1.31785  3.16424
EG.5/JN.1:10/10     0.78819 -1.11513  2.69150
EG.5/XBB.1.5:10/10  0.91151 -1.28986  3.11288
HV.1/JN.1:10/10     0.85376  0.63346  1.07407
HV.1/XBB.1.5:10/10  0.98734  0.72996  1.24472
JN.1/XBB.1.5:10/10  1.15646  1.06353  1.24939
```

```
drc::EDcomp(lmod,c(50,50),interval="delta")
```

```
Estimated ratios of effect doses

                   Estimate   Lower   Upper
EG.5/HV.1:50/50     0.97203 0.65240 1.29166
EG.5/JN.1:50/50     1.17185 0.91874 1.42496
EG.5/XBB.1.5:50/50  1.23159 0.96555 1.49763
HV.1/JN.1:50/50     1.20557 0.90574 1.50540
HV.1/XBB.1.5:50/50  1.26703 0.95189 1.58216
JN.1/XBB.1.5:50/50  1.05098 1.03030 1.07165
```

### GAM

```
gmod <- mgcv::gam(share~variant+s(t),data=var_data |> filter(variant%in%c("JN.1","XBB.1.5")))
summary(gmod)
```

```
Family: gaussian 
Link function: identity 

Formula:
share ~ variant + s(t)

Parametric coefficients:
               Estimate Std. Error t value Pr(>|t|)    
(Intercept)    0.266103   0.008274  32.160  < 2e-16 ***
variantXBB.1.5 0.036683   0.011558   3.174  0.00564 ** 
---
Signif. codes:  0 '***' 0.001 '**' 0.01 '*' 0.05 '.' 0.1 ' ' 1

Approximate significance of smooth terms:
       edf Ref.df     F p-value    
s(t) 6.262   7.43 440.9  <2e-16 ***
---
Signif. codes:  0 '***' 0.001 '**' 0.01 '*' 0.05 '.' 0.1 ' ' 1

R-sq.(adj) =  0.993   Deviance explained = 99.5%
GCV = 0.0012076  Scale est. = 0.00080852  n = 25
```

## Region 5

```
r="5"
```

```
startdate <- as.Date("2023-09-02")

variant_int <- "JN.1"

var_data_jn1 <- covid |> 
  filter(usa_or_hhsregion==r) |> 
  filter(variant %in% c(variant_int)) |> 
  mutate(t = as.numeric(week-startdate)) |> 
  arrange(t)

var_data_jn1 |> dplyr::select(t,share) |>slice_max(share,n=1) -> maxshare

maxtime <- maxshare[1] |> as.numeric()

var_data_jn1 <- var_data_jn1 |> 
  filter(between(t,0,maxtime))

startdate <- as.Date("2022-10-15")

variant_int <- "XBB.1.5"

var_data_xbb <- covid |> 
  filter(usa_or_hhsregion==r) |> 
  filter(variant %in% c(variant_int)) |> 
  mutate(t = as.numeric(week-startdate)) |> 
  arrange(t)

var_data_xbb |> dplyr::select(t,share) |>slice_max(share,n=1) -> maxshare

maxtime <- maxshare[1] |> as.numeric()

var_data_xbb <- var_data_xbb |> 
  filter(between(t,0,maxtime))

startdate <- as.Date("2023-06-10")

variant_int <- "HV.1"


var_data_ba <- covid |> 
  filter(usa_or_hhsregion==r) |> 
  filter(variant %in% c(variant_int)) |> 
  mutate(t = as.numeric(week-startdate)) |> 
  arrange(t)

var_data_ba |> dplyr::select(t,share) |>slice_max(share,n=1) -> maxshare

maxtime <- maxshare[1] |> as.numeric()

var_data_ba <- var_data_ba |> 
  filter(between(t,0,maxtime))

startdate <- as.Date("2023-03-18")

variant_int <- "EG.5"

var_data_eg <- covid |> 
  filter(usa_or_hhsregion==r) |> 
  filter(variant %in% c(variant_int)) |> 
  mutate(t = as.numeric(week-startdate)) |> 
  arrange(t)

var_data_eg |> dplyr::select(t,share) |>slice_max(share,n=1) -> maxshare

maxtime <- maxshare[1] |> as.numeric()

var_data_eg <- var_data_eg |> 
  filter(between(t,0,maxtime))


var_data <- bind_rows(var_data_jn1,var_data_xbb,var_data_ba,var_data_eg)

lmod <- drc::drm(share~t,as.factor(variant),fct=drc::L.5(),data=var_data)
```

```
drc::EDcomp(lmod,c(10,10),interval="delta")
```

```
Estimated ratios of effect doses

                   Estimate   Lower   Upper
EG.5/HV.1:10/10     1.13588 0.93234 1.33941
EG.5/JN.1:10/10     1.01369 0.90889 1.11849
EG.5/XBB.1.5:10/10  1.05099 0.94675 1.15523
HV.1/JN.1:10/10     0.89243 0.75188 1.03298
HV.1/XBB.1.5:10/10  0.92526 0.78206 1.06846
JN.1/XBB.1.5:10/10  1.03679 0.98462 1.08896
```

```
drc::EDcomp(lmod,c(50,50),interval="delta")
```

```
Estimated ratios of effect doses

                   Estimate   Lower   Upper
EG.5/HV.1:50/50     0.90254 0.72275 1.08234
EG.5/JN.1:50/50     1.04392 0.98232 1.10552
EG.5/XBB.1.5:50/50  1.15475 1.09594 1.21357
HV.1/JN.1:50/50     1.15664 0.93050 1.38279
HV.1/XBB.1.5:50/50  1.27945 1.03222 1.52667
JN.1/XBB.1.5:50/50  1.10617 1.06889 1.14345
```

### GAM

```
gmod <- mgcv::gam(share~variant+s(t),data=var_data |> filter(variant%in%c("JN.1","XBB.1.5")))
summary(gmod)
```

```
Family: gaussian 
Link function: identity 

Formula:
share ~ variant + s(t)

Parametric coefficients:
               Estimate Std. Error t value Pr(>|t|)    
(Intercept)     0.21072    0.01177  17.900 2.01e-12 ***
variantXBB.1.5  0.04932    0.01665   2.963  0.00876 ** 
---
Signif. codes:  0 '***' 0.001 '**' 0.01 '*' 0.05 '.' 0.1 ' ' 1

Approximate significance of smooth terms:
       edf Ref.df     F p-value    
s(t) 5.094  6.199 207.4  <2e-16 ***
---
Signif. codes:  0 '***' 0.001 '**' 0.01 '*' 0.05 '.' 0.1 ' ' 1

R-sq.(adj) =  0.983   Deviance explained = 98.7%
GCV = 0.0023609  Scale est. = 0.001663  n = 24
```

## Region 6

```
r="6"
```

```
startdate <- as.Date("2023-09-02")

variant_int <- "JN.1"

var_data_jn1 <- covid |> 
  filter(usa_or_hhsregion==r) |> 
  filter(variant %in% c(variant_int)) |> 
  mutate(t = as.numeric(week-startdate)) |> 
  arrange(t)

var_data_jn1 |> dplyr::select(t,share) |>slice_max(share,n=1) -> maxshare

maxtime <- maxshare[1] |> as.numeric()

var_data_jn1 <- var_data_jn1 |> 
  filter(between(t,0,maxtime))

startdate <- as.Date("2022-10-15")

variant_int <- "XBB.1.5"

var_data_xbb <- covid |> 
  filter(usa_or_hhsregion==r) |> 
  filter(variant %in% c(variant_int)) |> 
  mutate(t = as.numeric(week-startdate)) |> 
  arrange(t)

var_data_xbb |> dplyr::select(t,share) |>slice_max(share,n=1) -> maxshare

maxtime <- maxshare[1] |> as.numeric()

var_data_xbb <- var_data_xbb |> 
  filter(between(t,0,maxtime))

startdate <- as.Date("2023-06-10")

variant_int <- "HV.1"


var_data_ba <- covid |> 
  filter(usa_or_hhsregion==r) |> 
  filter(variant %in% c(variant_int)) |> 
  mutate(t = as.numeric(week-startdate)) |> 
  arrange(t)

var_data_ba |> dplyr::select(t,share) |>slice_max(share,n=1) -> maxshare

maxtime <- maxshare[1] |> as.numeric()

var_data_ba <- var_data_ba |> 
  filter(between(t,0,maxtime))

startdate <- as.Date("2023-03-18")

variant_int <- "EG.5"

var_data_eg <- covid |> 
  filter(usa_or_hhsregion==r) |> 
  filter(variant %in% c(variant_int)) |> 
  mutate(t = as.numeric(week-startdate)) |> 
  arrange(t)

var_data_eg |> dplyr::select(t,share) |>slice_max(share,n=1) -> maxshare

maxtime <- maxshare[1] |> as.numeric()

var_data_eg <- var_data_eg |> 
  filter(between(t,0,maxtime))


var_data <- bind_rows(var_data_jn1,var_data_xbb,var_data_ba,var_data_eg)

lmod <- drc::drm(share~t,as.factor(variant),fct=drc::L.5(),data=var_data)
```

```
drc::EDcomp(lmod,c(10,10),interval="delta")
```

```
Estimated ratios of effect doses

                   Estimate   Lower   Upper
EG.5/HV.1:10/10     1.14390 0.65894 1.62887
EG.5/JN.1:10/10     1.03730 0.71154 1.36306
EG.5/XBB.1.5:10/10  1.12726 0.76674 1.48778
HV.1/JN.1:10/10     0.90681 0.64325 1.17036
HV.1/XBB.1.5:10/10  0.98545 0.69290 1.27800
JN.1/XBB.1.5:10/10  1.08673 0.99568 1.17777
```

```
drc::EDcomp(lmod,c(50,50),interval="delta")
```

```
Estimated ratios of effect doses

                   Estimate   Lower   Upper
EG.5/HV.1:50/50     0.99306 0.91401 1.07210
EG.5/JN.1:50/50     1.04329 0.97645 1.11013
EG.5/XBB.1.5:50/50  1.09547 1.02571 1.16523
HV.1/JN.1:50/50     1.05058 0.98707 1.11410
HV.1/XBB.1.5:50/50  1.10313 1.03689 1.16937
JN.1/XBB.1.5:50/50  1.05002 1.01109 1.08894
```

### GAM

```
gmod <- mgcv::gam(share~variant+s(t),data=var_data |> filter(variant%in%c("JN.1","XBB.1.5")))
summary(gmod)
```

```
Family: gaussian 
Link function: identity 

Formula:
share ~ variant + s(t)

Parametric coefficients:
               Estimate Std. Error t value Pr(>|t|)    
(Intercept)    0.255306   0.005059  50.467  < 2e-16 ***
variantXBB.1.5 0.028230   0.007363   3.834  0.00144 ** 
---
Signif. codes:  0 '***' 0.001 '**' 0.01 '*' 0.05 '.' 0.1 ' ' 1

Approximate significance of smooth terms:
       edf Ref.df    F p-value    
s(t) 6.823  7.937 1055  <2e-16 ***
---
Signif. codes:  0 '***' 0.001 '**' 0.01 '*' 0.05 '.' 0.1 ' ' 1

R-sq.(adj) =  0.997   Deviance explained = 99.8%
GCV = 0.00050613  Scale est. = 0.00032752  n = 25
```

## Region 7

```
r="7"
```

```
startdate <- as.Date("2023-09-02")

variant_int <- "JN.1"

var_data_jn1 <- covid |> 
  filter(usa_or_hhsregion==r) |> 
  filter(variant %in% c(variant_int)) |> 
  mutate(t = as.numeric(week-startdate)) |> 
  arrange(t)

var_data_jn1 |> dplyr::select(t,share) |>slice_max(share,n=1) -> maxshare

maxtime <- maxshare[1] |> as.numeric()

var_data_jn1 <- var_data_jn1 |> 
  filter(between(t,0,maxtime))

startdate <- as.Date("2022-10-15")

variant_int <- "XBB.1.5"

var_data_xbb <- covid |> 
  filter(usa_or_hhsregion==r) |> 
  filter(variant %in% c(variant_int)) |> 
  mutate(t = as.numeric(week-startdate)) |> 
  arrange(t)

var_data_xbb |> dplyr::select(t,share) |>slice_max(share,n=1) -> maxshare

maxtime <- maxshare[1] |> as.numeric()

var_data_xbb <- var_data_xbb |> 
  filter(between(t,0,maxtime))

startdate <- as.Date("2023-06-10")

variant_int <- "HV.1"


var_data_ba <- covid |> 
  filter(usa_or_hhsregion==r) |> 
  filter(variant %in% c(variant_int)) |> 
  mutate(t = as.numeric(week-startdate)) |> 
  arrange(t)

var_data_ba |> dplyr::select(t,share) |>slice_max(share,n=1) -> maxshare

maxtime <- maxshare[1] |> as.numeric()

var_data_ba <- var_data_ba |> 
  filter(between(t,0,maxtime))

startdate <- as.Date("2023-03-18")

variant_int <- "EG.5"

var_data_eg <- covid |> 
  filter(usa_or_hhsregion==r) |> 
  filter(variant %in% c(variant_int)) |> 
  mutate(t = as.numeric(week-startdate)) |> 
  arrange(t)

var_data_eg |> dplyr::select(t,share) |>slice_max(share,n=1) -> maxshare

maxtime <- maxshare[1] |> as.numeric()

var_data_eg <- var_data_eg |> 
  filter(between(t,0,maxtime))


var_data <- bind_rows(var_data_jn1,var_data_xbb,var_data_ba,var_data_eg)

lmod <- drc::drm(share~t,as.factor(variant),fct=drc::L.5(),data=var_data)
```

```
drc::EDcomp(lmod,c(10,10),interval="delta")
```

```
Estimated ratios of effect doses

                   Estimate   Lower   Upper
EG.5/HV.1:10/10     1.10197 0.61981 1.58412
EG.5/JN.1:10/10     0.91587 0.59583 1.23590
EG.5/XBB.1.5:10/10  0.86350 0.57215 1.15485
HV.1/JN.1:10/10     0.83112 0.57554 1.08671
HV.1/XBB.1.5:10/10  0.78360 0.55340 1.01380
JN.1/XBB.1.5:10/10  0.94282 0.82001 1.06563
```

```
drc::EDcomp(lmod,c(50,50),interval="delta")
```

```
Estimated ratios of effect doses

                   Estimate   Lower   Upper
EG.5/HV.1:50/50     0.99592 0.78970 1.20214
EG.5/JN.1:50/50     0.90361 0.84014 0.96709
EG.5/XBB.1.5:50/50  0.92638 0.86235 0.99041
HV.1/JN.1:50/50     0.90731 0.72850 1.08612
HV.1/XBB.1.5:50/50  0.93017 0.74723 1.11311
JN.1/XBB.1.5:50/50  1.02519 0.99730 1.05309
```

### GAM

```
gmod <- mgcv::gam(share~variant+s(t),data=var_data |> filter(variant%in%c("JN.1","XBB.1.5")))
summary(gmod)
```

```
Family: gaussian 
Link function: identity 

Formula:
share ~ variant + s(t)

Parametric coefficients:
               Estimate Std. Error t value Pr(>|t|)    
(Intercept)    0.282902   0.009256  30.564   <2e-16 ***
variantXBB.1.5 0.036816   0.012947   2.843   0.0109 *  
---
Signif. codes:  0 '***' 0.001 '**' 0.01 '*' 0.05 '.' 0.1 ' ' 1

Approximate significance of smooth terms:
       edf Ref.df     F p-value    
s(t) 7.258  8.279 341.6  <2e-16 ***
---
Signif. codes:  0 '***' 0.001 '**' 0.01 '*' 0.05 '.' 0.1 ' ' 1

R-sq.(adj) =  0.991   Deviance explained = 99.4%
GCV = 0.0016683  Scale est. = 0.0010963  n = 27
```

## Region 8

```
r="8"
```

```
startdate <- as.Date("2023-09-02")

variant_int <- "JN.1"

var_data_jn1 <- covid |> 
  filter(usa_or_hhsregion==r) |> 
  filter(variant %in% c(variant_int)) |> 
  mutate(t = as.numeric(week-startdate)) |> 
  arrange(t)

var_data_jn1 |> dplyr::select(t,share) |>slice_max(share,n=1) -> maxshare

maxtime <- maxshare[1] |> as.numeric()

var_data_jn1 <- var_data_jn1 |> 
  filter(between(t,0,maxtime))

startdate <- as.Date("2022-10-15")

variant_int <- "XBB.1.5"

var_data_xbb <- covid |> 
  filter(usa_or_hhsregion==r) |> 
  filter(variant %in% c(variant_int)) |> 
  mutate(t = as.numeric(week-startdate)) |> 
  arrange(t)

var_data_xbb |> dplyr::select(t,share) |>slice_max(share,n=1) -> maxshare

maxtime <- maxshare[1] |> as.numeric()

var_data_xbb <- var_data_xbb |> 
  filter(between(t,0,maxtime))

startdate <- as.Date("2023-06-10")

variant_int <- "HV.1"


var_data_ba <- covid |> 
  filter(usa_or_hhsregion==r) |> 
  filter(variant %in% c(variant_int)) |> 
  mutate(t = as.numeric(week-startdate)) |> 
  arrange(t)

var_data_ba |> dplyr::select(t,share) |>slice_max(share,n=1) -> maxshare

maxtime <- maxshare[1] |> as.numeric()

var_data_ba <- var_data_ba |> 
  filter(between(t,0,maxtime))

startdate <- as.Date("2023-03-18")

variant_int <- "EG.5"

var_data_eg <- covid |> 
  filter(usa_or_hhsregion==r) |> 
  filter(variant %in% c(variant_int)) |> 
  mutate(t = as.numeric(week-startdate)) |> 
  arrange(t)

var_data_eg |> dplyr::select(t,share) |>slice_max(share,n=1) -> maxshare

maxtime <- maxshare[1] |> as.numeric()

var_data_eg <- var_data_eg |> 
  filter(between(t,0,maxtime))


var_data <- bind_rows(var_data_jn1,var_data_xbb,var_data_ba,var_data_eg)

lmod <- drc::drm(share~t,as.factor(variant),fct=drc::L.5(),data=var_data)
```

```
drc::EDcomp(lmod,c(10,10),interval="delta")
```

```
Estimated ratios of effect doses

                   Estimate   Lower   Upper
EG.5/HV.1:10/10     0.85126 0.37339 1.32914
EG.5/JN.1:10/10     0.79576 0.39702 1.19450
EG.5/XBB.1.5:10/10  0.86332 0.43166 1.29497
HV.1/JN.1:10/10     0.93480 0.69075 1.17886
HV.1/XBB.1.5:10/10  1.01416 0.75151 1.27682
JN.1/XBB.1.5:10/10  1.08490 1.02533 1.14446
```

```
drc::EDcomp(lmod,c(50,50),interval="delta")
```

```
Estimated ratios of effect doses

                   Estimate   Lower   Upper
EG.5/HV.1:50/50     0.81643 0.53402 1.09883
EG.5/JN.1:50/50     0.99756 0.93687 1.05825
EG.5/XBB.1.5:50/50  1.12903 1.06631 1.19175
HV.1/JN.1:50/50     1.22186 0.80272 1.64100
HV.1/XBB.1.5:50/50  1.38290 0.90976 1.85604
JN.1/XBB.1.5:50/50  1.13180 1.09403 1.16956
```

### GAM

```
gmod <- mgcv::gam(share~variant+s(t),data=var_data |> filter(variant%in%c("JN.1","XBB.1.5")))
summary(gmod)
```

```
Family: gaussian 
Link function: identity 

Formula:
share ~ variant + s(t)

Parametric coefficients:
               Estimate Std. Error t value Pr(>|t|)    
(Intercept)     0.22269    0.01479  15.055 2.93e-11 ***
variantXBB.1.5  0.06064    0.02092   2.899  0.00999 ** 
---
Signif. codes:  0 '***' 0.001 '**' 0.01 '*' 0.05 '.' 0.1 ' ' 1

Approximate significance of smooth terms:
       edf Ref.df   F p-value    
s(t) 5.003  6.097 151  <2e-16 ***
---
Signif. codes:  0 '***' 0.001 '**' 0.01 '*' 0.05 '.' 0.1 ' ' 1

R-sq.(adj) =  0.976   Deviance explained = 98.2%
GCV = 0.0037071  Scale est. = 0.0026254  n = 24
```

## Region 9

```
r="9"
```

```
startdate <- as.Date("2023-09-02")

variant_int <- "JN.1"

var_data_jn1 <- covid |> 
  filter(usa_or_hhsregion==r) |> 
  filter(variant %in% c(variant_int)) |> 
  mutate(t = as.numeric(week-startdate)) |> 
  arrange(t)

var_data_jn1 |> dplyr::select(t,share) |>slice_max(share,n=1) -> maxshare

maxtime <- maxshare[1] |> as.numeric()

var_data_jn1 <- var_data_jn1 |> 
  filter(between(t,0,maxtime))

startdate <- as.Date("2022-10-15")

variant_int <- "XBB.1.5"

var_data_xbb <- covid |> 
  filter(usa_or_hhsregion==r) |> 
  filter(variant %in% c(variant_int)) |> 
  mutate(t = as.numeric(week-startdate)) |> 
  arrange(t)

var_data_xbb |> dplyr::select(t,share) |>slice_max(share,n=1) -> maxshare

maxtime <- maxshare[1] |> as.numeric()

var_data_xbb <- var_data_xbb |> 
  filter(between(t,0,maxtime))

startdate <- as.Date("2023-06-10")

variant_int <- "HV.1"


var_data_ba <- covid |> 
  filter(usa_or_hhsregion==r) |> 
  filter(variant %in% c(variant_int)) |> 
  mutate(t = as.numeric(week-startdate)) |> 
  arrange(t)

var_data_ba |> dplyr::select(t,share) |>slice_max(share,n=1) -> maxshare

maxtime <- maxshare[1] |> as.numeric()

var_data_ba <- var_data_ba |> 
  filter(between(t,0,maxtime))

startdate <- as.Date("2023-03-18")

variant_int <- "EG.5"

var_data_eg <- covid |> 
  filter(usa_or_hhsregion==r) |> 
  filter(variant %in% c(variant_int)) |> 
  mutate(t = as.numeric(week-startdate)) |> 
  arrange(t)

var_data_eg |> dplyr::select(t,share) |>slice_max(share,n=1) -> maxshare

maxtime <- maxshare[1] |> as.numeric()

var_data_eg <- var_data_eg |> 
  filter(between(t,0,maxtime))


var_data <- bind_rows(var_data_jn1,var_data_xbb,var_data_ba,var_data_eg)

lmod <- drc::drm(share~t,as.factor(variant),fct=drc::L.5(),data=var_data)
```

### GAM

```
gmod <- mgcv::gam(share~variant+s(t),data=var_data |> filter(variant%in%c("JN.1","XBB.1.5")))
summary(gmod)
```

```
Family: gaussian 
Link function: identity 

Formula:
share ~ variant + s(t)

Parametric coefficients:
               Estimate Std. Error t value Pr(>|t|)    
(Intercept)     0.27687    0.01111   24.92 4.69e-15 ***
variantXBB.1.5 -0.03909    0.01551   -2.52   0.0218 *  
---
Signif. codes:  0 '***' 0.001 '**' 0.01 '*' 0.05 '.' 0.1 ' ' 1

Approximate significance of smooth terms:
       edf Ref.df     F p-value    
s(t) 5.603  6.765 235.1  <2e-16 ***
---
Signif. codes:  0 '***' 0.001 '**' 0.01 '*' 0.05 '.' 0.1 ' ' 1

R-sq.(adj) =  0.985   Deviance explained = 98.9%
GCV = 0.0020975  Scale est. = 0.0014596  n = 25
```

## Region 10

```
r="10"
```

```
startdate <- as.Date("2023-09-02")

variant_int <- "JN.1"

var_data_jn1 <- covid |> 
  filter(usa_or_hhsregion==r) |> 
  filter(variant %in% c(variant_int)) |> 
  mutate(t = as.numeric(week-startdate)) |> 
  arrange(t)

var_data_jn1 |> dplyr::select(t,share) |>slice_max(share,n=1) -> maxshare

maxtime <- maxshare[1] |> as.numeric()

var_data_jn1 <- var_data_jn1 |> 
  filter(between(t,0,maxtime))

startdate <- as.Date("2022-10-15")

variant_int <- "XBB.1.5"

var_data_xbb <- covid |> 
  filter(usa_or_hhsregion==r) |> 
  filter(variant %in% c(variant_int)) |> 
  mutate(t = as.numeric(week-startdate)) |> 
  arrange(t)

var_data_xbb |> dplyr::select(t,share) |>slice_max(share,n=1) -> maxshare

maxtime <- maxshare[1] |> as.numeric()

var_data_xbb <- var_data_xbb |> 
  filter(between(t,0,maxtime))

startdate <- as.Date("2023-06-10")

variant_int <- "HV.1"


var_data_ba <- covid |> 
  filter(usa_or_hhsregion==r) |> 
  filter(variant %in% c(variant_int)) |> 
  mutate(t = as.numeric(week-startdate)) |> 
  arrange(t)

var_data_ba |> dplyr::select(t,share) |>slice_max(share,n=1) -> maxshare

maxtime <- maxshare[1] |> as.numeric()

var_data_ba <- var_data_ba |> 
  filter(between(t,0,maxtime))

startdate <- as.Date("2023-03-18")

variant_int <- "EG.5"

var_data_eg <- covid |> 
  filter(usa_or_hhsregion==r) |> 
  filter(variant %in% c(variant_int)) |> 
  mutate(t = as.numeric(week-startdate)) |> 
  arrange(t)

var_data_eg |> dplyr::select(t,share) |>slice_max(share,n=1) -> maxshare

maxtime <- maxshare[1] |> as.numeric()

var_data_eg <- var_data_eg |> 
  filter(between(t,0,maxtime))


var_data <- bind_rows(var_data_jn1,var_data_xbb,var_data_ba,var_data_eg)

lmod <- drc::drm(share~t,as.factor(variant),fct=drc::L.5(),data=var_data)
```

```
drc::EDcomp(lmod,c(10,10),interval="delta")
```

```
Warning in sqrt(t(dSIval) %*% varMat %*% dSIval): NaNs produced
Warning in sqrt(t(dSIval) %*% varMat %*% dSIval): NaNs produced
Warning in sqrt(t(dSIval) %*% varMat %*% dSIval): NaNs produced
```

```
Estimated ratios of effect doses

                   Estimate   Lower   Upper
EG.5/HV.1:10/10     0.67990     NaN     NaN
EG.5/JN.1:10/10     1.01632     NaN     NaN
EG.5/XBB.1.5:10/10  0.98845     NaN     NaN
HV.1/JN.1:10/10     1.49481 0.75008 2.23954
HV.1/XBB.1.5:10/10  1.45382 0.72587 2.18178
JN.1/XBB.1.5:10/10  0.97258 0.84233 1.10283
```

```
drc::EDcomp(lmod,c(50,50),interval="delta")
```

```
Warning in sqrt(t(dSIval) %*% varMat %*% dSIval): NaNs produced
Warning in sqrt(t(dSIval) %*% varMat %*% dSIval): NaNs produced
Warning in sqrt(t(dSIval) %*% varMat %*% dSIval): NaNs produced
```

```
Estimated ratios of effect doses

                   Estimate   Lower   Upper
EG.5/HV.1:50/50     0.76952     NaN     NaN
EG.5/JN.1:50/50     1.59127     NaN     NaN
EG.5/XBB.1.5:50/50  1.56466     NaN     NaN
HV.1/JN.1:50/50     2.06788 1.19572 2.94003
HV.1/XBB.1.5:50/50  2.03330 1.17515 2.89145
JN.1/XBB.1.5:50/50  0.98328 0.94886 1.01770
```

### GAM

```
gmod <- mgcv::gam(share~variant+s(t),data=var_data |> filter(variant%in%c("JN.1","XBB.1.5")))
summary(gmod)
```

```
Family: gaussian 
Link function: identity 

Formula:
share ~ variant + s(t)

Parametric coefficients:
                Estimate Std. Error t value Pr(>|t|)    
(Intercept)     0.325528   0.005831  55.823  < 2e-16 ***
variantXBB.1.5 -0.031487   0.008694  -3.622  0.00189 ** 
---
Signif. codes:  0 '***' 0.001 '**' 0.01 '*' 0.05 '.' 0.1 ' ' 1

Approximate significance of smooth terms:
       edf Ref.df     F p-value    
s(t) 7.558  8.474 799.5  <2e-16 ***
---
Signif. codes:  0 '***' 0.001 '**' 0.01 '*' 0.05 '.' 0.1 ' ' 1

R-sq.(adj) =  0.996   Deviance explained = 99.7%
GCV = 0.00075302  Scale est. = 0.00049598  n = 28
```
