# Supplementary material for: Emergence to dominance: Estimating time to dominance of SARS-CoV-2 variants using nonlinear statistical models
Source: PLoS One. 2025 Apr 2;20(4):e0311459. doi: 10.1371/journal.pone.0311459 (PMC11964452; doi:10.1371/journal.pone.0311459)
Supplement: Attachment — Submitted filename: Response to Reviewers.docx [file pone.0311459.s003.docx]

**To the Reviewer:**

**Thank you for providing us your comments and insights for improvement of the study. Please see our responses to your comments in boldface. If you require anything else, please feel free to reach out to me via email:** [**miguel.fudolig@unlv.edu**](mailto:miguel.fudolig@unlv.edu) **or through the editor.**

Reviewer #1: The authors have tested the efficiency of Weibull models, logistic models and GAMs in the retrospective analysis of the emergence of the XBB.1.5 and JN.1 variants in the US. The accurate prediction of the fate of an emerging variant is important in the management of the public health response to pathogens, and this paper adds to that work. Here the focus is retrospective and phenomenological, attempting to see how well different non-mechanistic models were able to fit the curve after the fact, estimating both the shape of the curve and the time to dominance of the variant. The approach taken here seems reasonable, and the work answers the question asked.

An interesting question that could be asked with this data, that the authors do not attempt to address with this work, but I think would considerably add to the general interest of the manuscript is how well the different methods manage to estimate the time to dominance prior to this time, as this is issue that faces public health authorities looking forward in time attempting to plan a response. (Perhaps this could be done by adding data points in turn to see how the estimate improves with each extra days' worth of data).

**This is a valid point to raise. Estimating the time to dominance prior to the time to dominance changes the focus from estimation to prediction. While we can use the models to extrapolate and predict the time to dominance that way, it could present some dangerous consequences if done blindly. For instance:**

- **There are VOIs that were expected to dominate in the population but were unable to do so because of the emergence of another highly infectious variant/subvariant. While the models can predict a time to dominance for these variants, the prediction might be incorrect due to reasons that cannot be accounted for using a phenomenological reason.**
- **To address your suggestion, we can add data points to improve TTD estimates. However, this is tough to expect from real data because of the limitations on the time it takes to perform genomic sequencing.**

**Both these problems could be circumvented by incorporating multi-strain simulations and testing the prediction and effect of lower temporal resolution between tests, but this is currently outside the scope of the paper. We included a detailed discussion of these points in Lines 289-303 and added a recommendation in our conclusions in Lines 365-366.**

Additionally, it would be nice if the authors could make the analysis code available as well as the data.

**Thank you for this suggestion. I have uploaded an HTML file of the R code and output. The data can be downloaded from the CDC website and I have also uploaded it as part of the supplementary material.**

Beyond this I have some minor comments that should be addressed before publication:

Line 12-13: While it is true that the RNA polymerase is low fidelity, SARS-CoV-2 actually has a complicated proofreading mechanism to ensure genome stability, so this sentence should be amended. See: <https://www.pnas.org/doi/pdf/10.1073/pnas.2106379119> **Thank you for this comment. We have edited this to reflect the lack of proofreading mechanisms to ensure high fidelity (Line 13-14)**

Lines 15-21: This is a direct restatement of what is contained in lines 8-15. Did you meant to say this twice? **No. This was an error. We have removed Lines 15-21 in the edited manuscript. Thank you very much.**

Lines 28-32: I would avoid saying that variants are "from" these countries. In some cases that will be true, but in others it may just represent where they were first detected, and it's impossible to tell which is which as the moment of emergence in unobserved. **Thank you very much for this input. We recognize how this language could be harmful and misleading. We have edited “from” to “first detected in” and was applied to all listed variants. (Lines 24-27).**

Line 143: Is there a missing word at the end of this sentence? **Yes. It was a LaTeX call to the section “Estimating Time to Dominance” that yielded a blank because section numbers were deleted. The section name was included in the revision. Thank you for bringing this oversight to our attention. (Line 140)**

Line 179: ED50 is not defined. **Thank you for noticing this. We have included the definition of ED50. (Lines 176-178)**
